# Targeted siRNA nanocarrier: a platform technology for cancer treatment

**DOI:** 10.1038/s41388-022-02241-w

**Published:** 2022-02-26

**Authors:** Nicole Bäumer, Jessica Tiemann, Annika Scheller, Theresa Meyer, Lisa Wittmann, Matias Ezequiel Gutierrez Suburu, Lilo Greune, Matthias Peipp, Neele Kellmann, Annika Gumnior, Caroline Brand, Wolfgang Hartmann, Claudia Rossig, Carsten Müller-Tidow, Dario Neri, Cristian A. Strassert, Christian Rüter, Petra Dersch, Georg Lenz, H. Phillip Koeffler, Wolfgang E. Berdel, Sebastian Bäumer

**Affiliations:** 1 Department of Medicine A, Hematology/Oncology, University of Münster, Münster, Germany; 2 Institute of Infectiology—Center for Molecular Biology of Inflammation (ZMBE), University of Münster, Münster, Germany; 3 Institute of Inorganic and Analytical Chemistry, University of Münster, Münster, Germany; 4 Division of Stem Cell Transplantation and Immunotherapy, Christian-Albrechts-University, Kiel, Germany; 5 Gerhard-Domagk Institute for Pathology, University of Münster, Münster, Germany; 6 Department of Pediatric Hematology and Oncology, University Children’s Hospital Münster, Münster, Germany; 7 Department of Medicine V, University of Heidelberg, Heidelberg, Germany; 8 Institute of Pharmaceutical Sciences, ETH Zurich, Zurich, Switzerland; 9 Cedars Sinai Medical Center, University of California, Los Angeles, CA USA

**Keywords:** Targeted therapies, Non-small-cell lung cancer, Sarcoma, Drug delivery

## Abstract

The small arginine-rich protein protamine condenses complete genomic DNA into the sperm head. Here, we applied its high RNA binding capacity for spontaneous electrostatic assembly of therapeutic nanoparticles decorated with tumour-cell-specific antibodies for efficiently targeting siRNA. Fluorescence microscopy and DLS measurements of these nanocarriers revealed the formation of a vesicular architecture that requires presence of antibody-protamine, defined excess of free SMCC-protamine, and anionic siRNA to form. Only these complex nanoparticles were efficient in the treatment of non-small-cell lung cancer (NSCLC) xenograft models, when the oncogene KRAS was targeted via EGFR-mediated delivery. To show general applicability, we used the modular platform for IGF1R-positive Ewing sarcomas. Anti-IGR1R-antibodies were integrated into an antibody-protamine nanoparticle with an siRNA specifically against the oncogenic translocation product EWS/FLI1. Using these nanoparticles, EWS/FLI1 knockdown blocked in vitro and in vivo growth of Ewing sarcoma cells. We conclude that these antibody-protamine-siRNA nanocarriers provide a novel platform technology to specifically target different cell types and yet undruggable targets in cancer therapy by RNAi.

## Introduction

In the last years, the directed transfer of nucleic acids for therapeutic purposes has attracted much attention. For gene therapy, by far the majority of approaches rely on viral transfer by lentiviruses or adenoviruses to the cells of interest. The transfer of RNA molecules is more complex.

High expectations were raised concerning the use of small interfering RNAs (siRNA) against gain-of-function gene products such as oncogenes in malignant neoplasia. However, therapeutic use of siRNAs was always compromised by their instability and missing cell-specific carrier systems. Thus, the development of an efficient siRNA nanocarrier is a major goal to make use of RNAi as a molecular therapeutic modality. To achieve this, we developed a technique for antibody-mediated siRNA therapy comprised of electrostatic nanocarriers consisting of antibody-protamine, protamine and electrostatically bound siRNA (α-P/siRNA/P).

Protamine is a small arginine-rich protein that displaces histones from chromatin during spermatogenesis leading to a specific DNA density that approaches that of a crystalline state. Clinically used as a heparin antidote [1, 2], protamine strongly coordinates this sulphated polysaccharide-anion [3]. In the 1960s, this strong nucleic acid coordination capacity led to the discovery that the addition of basic proteins such as protamine enhances the uptake of RNA by tumour cells in culture [4]. The RNA condensation effect by protamine has also been shown to promote resistance to degradation of RNA by nucleases [5].

Here, we employed the high RNA binding capacity of protamine to form a therapeutic, systemically applicable, targeted nanoparticle functioning as nanocarrier of tumour-cell-specific siRNA for delivery into tumour cells. In previous publications, we showed that the siRNA in this complex is stabilised by the tight interaction with protamine, internalises into EGFR-positive cells and exerts therapeutic anti-cancer activity in vitro and in vivo [6–8].

During the process of further characterisation of our antibody-protamine-siRNA complexes, we unexpectedly detected that unbound bifunctional crosslinker sulfo-SMCC (SMCC)-protamine is an indispensable component of our targeting complex. Further analysis revealed that our conjugates do not consist of a linear molecule antibody > SMCC-protamine > siRNA, as suggested by others before [9], but rather of a complex spheroid vesicle structure comprising a nanoparticle that binds the siRNA.

To apply this strategy to target two different tumour entities, non-small cell lung cancer (NSCLC) as well as Ewing sarcoma, a mesenchymal paediatric bone cancer, we have used our nanocarrier system with protamine-bound siRNA linked to the cancer cell-specific anti-EGFR-antibody cetuximab [6, 8] and the anti-insulin-like growth factor 1 receptor (IGF1R) antibodies cixutumumab and teprotumumab, respectively. Nanocarriers with these antibodies deliver siRNA to the intended cancer cells, bind to their respective receptors, internalise siRNA in a receptor-dependent fashion and exert strong anti-cancer activity against both types of tumours in vitro and in vivo.

## Results

### siRNA targeting by antibody-protamine conjugates requires a specific conjugation protocol

**Fig. 1:**
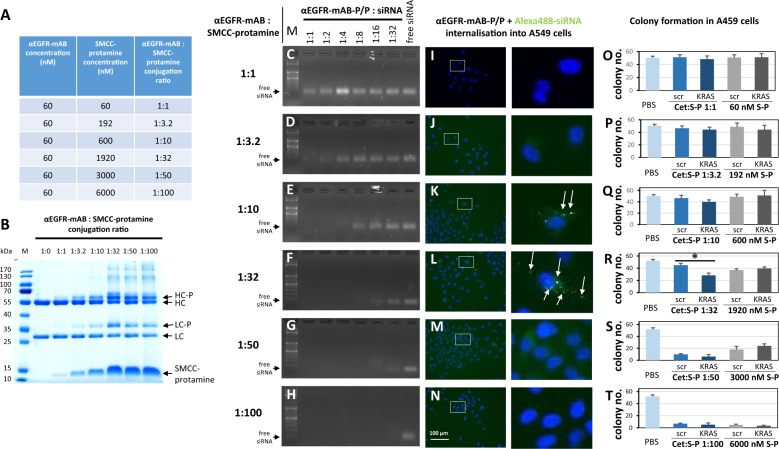
Attributes of effective anti-EGFR-mAB-protamine conjugation ratios. **A** Concentrations tested and resulting molar ratios of anti-(α)EGFR antibody (αEGFR-mAB) cetuximab to SMCC-protamine for the effective conjugation of both components. **B** Coomassie-stained SDS-PAGE showing uncoupled αEGFR-mAB cetuximab compared to the conjugation products that were coupled as depicted in **A**. The formation of a protamine-conjugated heavy chain (HC-P) and light chain (LC-P) showed an optimum at a 1:32 conjugation ratio with no further increase at higher ratios. **C**–**H** Band-shift assays exhibiting siRNA binding capacity. **I**–**N** Internalisation of Alexa488-control-siRNA complexed by αEGFR-protamine and free SMCC-protamine (αEGFR-mAB-P/P) in A549 cells. Complexes of αEGFR-mAB-P/P transport Alexa488-siRNA into cells (left panel rectangles), with detailed magnifications (right panels). **O**–**T** Colony formation assays using the complexes analysed in C-H and I-N in EGFR-positive A549 cells. Significant effect of αEGFR-mAB-P/P transported KRAS siRNA effect in contrast to control scrambled (scr) siRNA is only seen in conjugate preparations with 1:32 molar ratio mAB to protamine (**R**). Conversely, lower ratios show ineffective binding of siRNA (**C**, **D**), do not internalise (**I**, **J**) and achieve no sufficient functional effect (**O**–**Q**), while preparations with higher molar excess of protamine-SMCC show toxicity independent of KRAS knockdown (**S**, **T**). A further selection of increments of coupling ratios between 1:20 to 1:40 were presented in Supplementary Fig. 2. Cet:S-P αEGFR-antibody cetuximab conjugated to SMCC-protamine at the indicated ratios, S-P SMCC-protamine. Mean ± SD of three independent experiments. Two-sided *t*-test, *p < 0.05.

An siRNA-carrier has to fulfil two essential requirements to serve as effective therapeutic agent: first, it has to bind siRNA cargo efficiently and prevent siRNA degradation, second it must bind to a cell determining and internalising moiety to deliver this complex to the intended tumour cells and internalise the therapeutic cargo. In order to optimise our antibody-protamine-siRNA-carrier system [6, 8], we tested different molecular ratios of anti (α)EGFR monoclonal antibody (mAB) cetuximab and SMCC-bound protamine. We conjugated molar ratios from 1:1 to a 1:100 (Fig. 1A and Supplementary Figs. 1 and 2) excess of SMCC-protamine over IgG and checked the gel-electrophoretic properties of the resulting conjugates (Fig. 1B and Supplementary Fig. 2B), their respective ability to bind siRNA (Fig. 1C–H and Supplementary Fig. 2C–F), and the internalisation of fluorescently tagged siRNA into EGFR-expressing NSCLC cells (Fig. 1I–N and Supplementary Fig. 2K–N). Only conjugates with a molar excess of 10–35 mol SMCC-protamine over IgG showed significant Alexa488-siRNA internalisation capacity (Fig. 1K, L and Supplementary Fig. 2K–N). None of these complexes could mediate Alexa488-siRNA transport into EGFR-negative cells such as the Ewing sarcoma cell line SK-N-MC (Supplementary Fig. 1A–F). Moreover, Alexa488-siRNA could neither be transported efficiently by free SMCC-protamine into SK-N-MC Ewing cells (Supplementary Fig. 1G–L) nor into A549 NSCLC cells (Supplementary Fig. 1M–R), which indicates that the internalisation depends on the specific interaction of the αEGFR-mAB moiety with the respective receptor rather than on unspecific uptake of SMCC-protamine-siRNA.

Next, we determined the functional impact of the different conjugation ratios on their ability to inhibit the driving oncogene KRAS. To this purpose, we treated NSCLC A549 cells with different αEGFR-antibody-protamine-complexes with control (scrambled, scr) vs. anti-KRAS siRNA, respectively, and cultured equal numbers of cells in soft agar for anchorage-independent colony growth indicative for tumourigenicity. Of note, only the 1:32 (Fig. 1R), the 1:35 and 1:40 conjugates (Supplementary Fig. 2Q, R) showed a functional impact of KRAS siRNA vs. control-siRNA with 50% less colony formation, compared to non-functional control-siRNA, whereas compositions with lower excess of 1:1 up to 1:25 showed no impact of KRAS siRNA on colony growth (Fig. 1O–Q and Supplementary Fig. 2O, P), and conjugation protocols exceeding the 1:32 ratio mediated unspecific toxicity of SMCC-protamine at very high concentrations exceeding 3000 nM, unrelated to the nature of the applied siRNA (Fig. 1S, T and Supplementary Fig. 2Q, R).

To this end, we conclude that the conjugation reaction between the αEGFR-mAB coupled to SMCC-protamine and a 32x excess of SMCC-protamine which is combined with 10 mol siRNA per mol of IgG-conjugate yields an optimal formulation to bind, transport, internalise and consequently liberate functionally effective siRNA to EGFR-expressing tumour cells.

### Transport of siRNA via antibody-protamine complexes requires the presence of free SMCC-protamine molecules

**Fig. 2:**
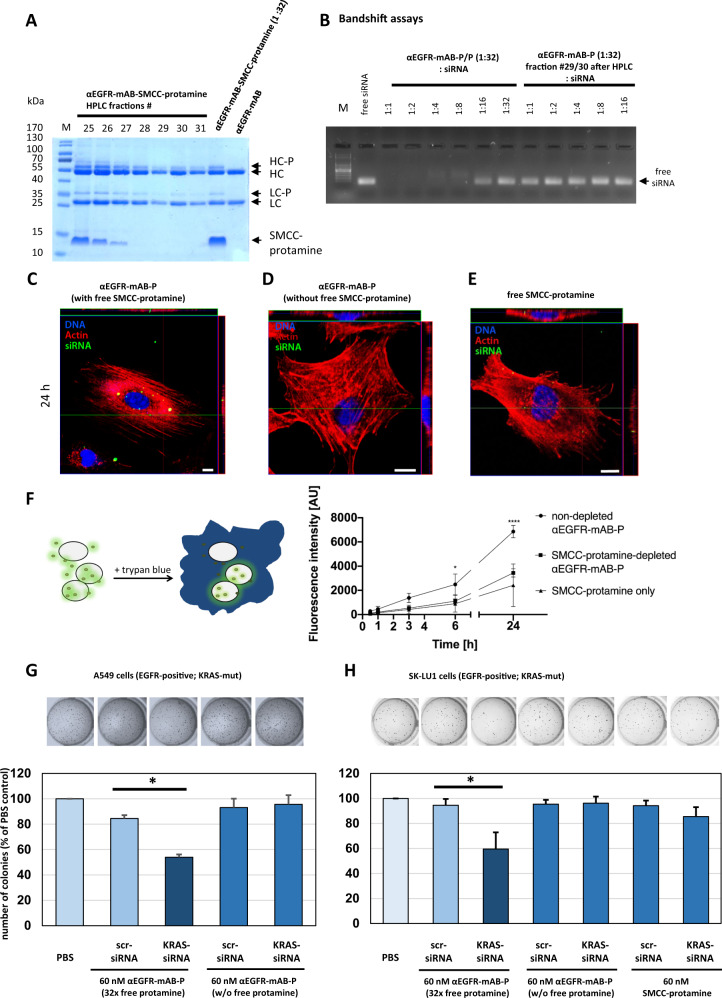
Anti-EGFR-mAB-protamine (αEGFR-mAB-P) conjugates do not bind and transport siRNA efficiently after depletion of free SMCC-protamine by HPLC. **A** Coomassie-stained SDS-PAGE showing αEGFR-mAB-P, αEGFR-mAB-P coupled with 32x SMCC-protamine and HPLC-fractions 25–31 of anti-EGFR-mAB coupled with 32x SMCC-protamine after depletion of unbound SMCC-protamine; HC heavy chain, LC light chain, -P SMCC-protamine. **B** Band-shift assays showing that non-SMCC-protamine-depleted αEGFR-mAB-P binds siRNA in a 1:8 to 1:16 molar ratio (left part), whereas the chromatographically depleted αEGFR-mAB-P does not bind siRNA. **C**–**E** Dynamics of internalisation of Alexa488-siRNA by confocal internalisation studies with depleted vs. non-depleted αEGFR-mAB-P with fluorescence-tagged siRNA on A549 NSCLC cells. Blue fluorescence for nuclei, red for actin, green for internalised Alexa488-siRNA. **C** Non-depleted αEGFR-mAB-P transports Alexa488-siRNA to A549 intracellular vesicles. Please note the cytoplasmic and perinuclear localisation of Alexa488-positive vesicles (compare also to Supplementary Fig. 5). The purification process leads to abolished internalisation of siRNA (**D**). SMCC-protamine alone does not work as an unspecific transfection agent (**E**). Scale bars 10 µm. **F** Dynamics of Alexa488-siRNA internalisation to A549 cells mediated by non-depleted αEGFR-mAB-P/P vs. SMCC-protamine-depleted αEGFR-mAB-P, controlled by SMCC-protamine only as carrier molecule in flow cytometric analysis (*n* = 3; error bars indicate SEM). Statistical significance was tested by two-way ANOVA with subsequent Tukey’s multiple comparison test (*p < 0.05, **p < 0.01, ***p < 0.001, ****p < 0.0001). Of note, only the non-SMCC-protamine-depleted conjugate presented a significant siRNA internalisation compared to SMCC-protamine-depleted and SMCC-protamine from 6 h into A549 cells. **G**, **H** Functional relevance of free SMCC-protamine in the reaction mixture. Non-depleted αEGFR-mAB-P-transported KRAS siRNA leads to significant reduction of colony formation in NSCLC A549 cells (**G**) and NSCLC SK-LU1 cells (**H**) in comparison to control (scrambled, scr) siRNA, whereas the depletion of free SMCC-protamine completely abolishes this effect. SMCC-protamine alone (without αEGFR-mAB) again is not able to efficiently transport and internalise KRAS siRNA seen by no effect in colony assay (**H**, right bars; Mean ± SD of three independent experiments. Two-sided *t*-test, *p < 0.05).

To translate siRNA-carrier production into clinical application according to good manufacturing practice (GMP), we intended to remove all excess free SMCC-protamine from the reaction mixture by preparative size exclusion chromatography or by protein G interaction chromatography (Fig. 2).

As shown in Fig. 2A, the 32:1 protamine-conjugated antibody was bound to the protein G matrix, the unbound SMCC-protamine eluted early and was followed by the purified IgG-protamine complex (Fig. 2, see fractions 29–31). Unexpectedly, this material, although protamine-conjugated, was not able to bind siRNA in a band-shift assay (Fig. 2B), whereas the non-purified mAB-protamine complex was binding siRNA at the usual 1:16 molar ratio. We further analysed the same two conjugate samples, the purified and thus SMCC-protamine depleted vs. non-purified and thus SMCC-protamine containing: First, it became evident that the SMCC-protamine depleted samples were not able to mediate an internalisation of Alexa488-tagged siRNA (Fig. 2D) in contrast to the non-purified, complete conjugate (Fig. 2C) with clear cytoplasmatic vesicular deposits seen in the *Z*-axis in confocal laser microscopic (CLS) analysis. On the other hand, SMCC-protamine alone with no attached targeting antibody cannot serve as an unspecific transfection reagent (Fig. 2E and Supplementary Figs. 1M–R and 5 for A549 and Supplementary Fig. 1G–L). Moreover, the targeting of functionally active siRNA is highly specific to EGFR-positive cells (Supplementary Fig. 4A).

Next, we tested the dynamics of Alexa488-siRNA internalisation to A549 cells mediated by the depleted vs. non-depleted carrier conjugates over a 24 h time frame analysed by flow cytometry (Fig. 2F). From 6 h on, the non-depleted αEGFR-mAB-protamine conjugate robustly and significantly internalised siRNA, whereas the protamine-depleted conjugate internalised much less siRNA, comparable to the background levels internalised by SMCC-protamine only. Of note, the flow cytometry assay detects the internalised Alexa488 signal exclusively, while all peripheral, non-internalised “sticky” signals connected to the cell membrane are quenched by trypan blue (Fig. 2F, left panel schematic overview) [10]. These results were validated by CLS microscopy of the same cells at the indicated time points (Supplementary Fig. 5), where only the non-depleted and antibody-targeted complex was able to internalise siRNA, whereas the protamine-depleted complex was incompetent of targeting (Supplementary Fig. 5, middle column) and the pure SMCC-protamine preparation without any antibody revealed some vesicular markings after 6 h of treatment that later disappeared (Supplementary Fig. 5, right column) and were non-functional (Fig. 1R).

Moreover, non-depleted αEGFR-mAB-P-transported KRAS siRNA led to significant reduction of colony formation in NSCLC A549 cells (Fig. 2G) and NSCLC SK-LU1 cells (Fig. 2H) in contrast to control-siRNA, whereas the depletion of free SMCC-protamine completely abolished this effect. SMCC-protamine alone again was not able to efficiently transport and internalise KRAS siRNA, as seen by no effect in colony assay (Fig. 2H, right bars).

### Antibody-protamine, free SMCC-protamine and siRNA form unexpected vesicular nanoparticles

**Fig. 3:**
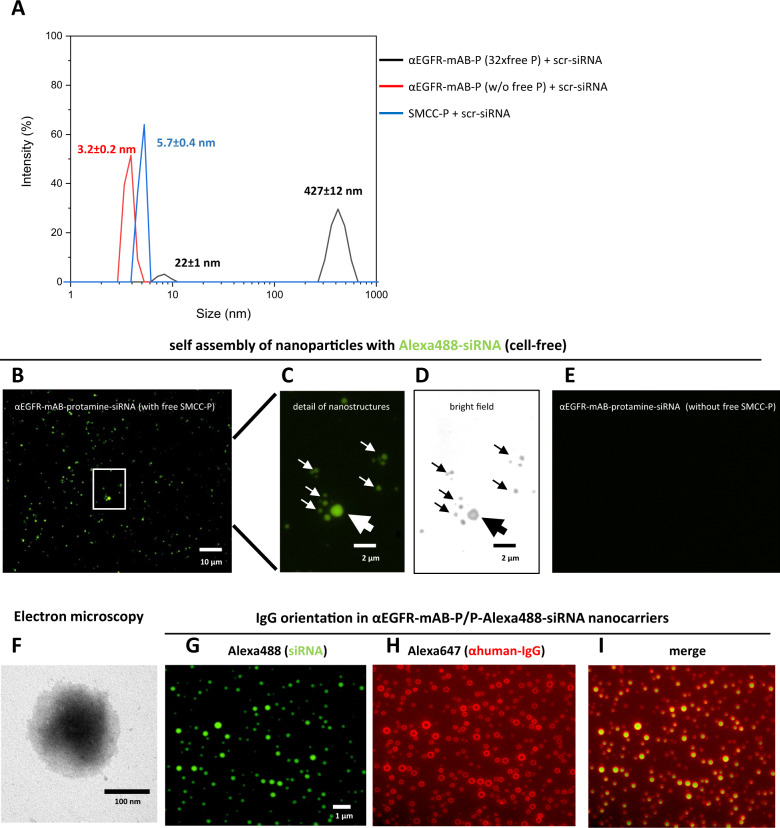
αEGFR-mAB-protamine (αEGFR-mAB-P) conjugates formed nanoparticles require free SMCC-protamine to form a stable complex with siRNA. **A** The 1:32 mAB-protamine conjugate mixture was depleted of excess free SMCC-protamine (free P) by protein G-affinity chromatography. Fractions without excess free SMCC-protamine were compared to fractions still containing excess free SMCC-protamine and the unconjugated SMCC-protamine (without αEGFR-mAB-P) in dynamic light scattering spectroscopy (DLS). Only fractions containing αEGFR-mAB-P, excess unconjugated SMCC-protamine and siRNA exhibited the ability to form larger nanostructures overnight confirmed by dynamic light scattering spectroscopy (black curves, particle size 427 ± 12 nm), but not SMCC-protamine-depleted (red curves, 3.2 nm), or preparations only consisting of free SMCC-protamine and control (scramble, scr) siRNA (blue curves, 5.7 nm) after 2 h of self-assembly. **B**–**D** Non-purified αEGFR-mAB-P conjugate preparations in complex with Alexa488-siRNA were mounted on glass slides and subjected to fluorescence microscopy. The particles detected in DLS analysis could be verified in microscopy in fluorescence (**B** and **C**) as well as bright field microscopy (**D**, same frame as in **C**). **E** αEGFR-mAB-P conjugate depleted from free SMCC-protamine in complex with Alexa488-siRNA were mounted on glass slides and subjected to fluorescence microscopy. No nanoparticles could be observed here. Also, formulations lacking αEGFR-mAB-P, consisting only of SMCC-protamine formed no such particles visible in microscopy (not shown). **F** αEGFR-mAB-P/P-scrm siRNA nanoparticles were left to form for 2 h and subjected to electron microscopy on copper grids by phospho-Wolfram negative staining. **G**–**I** αEGFR-mAB-P/P-Alexa488-siRNA nanocarriers formed for 2 h (**G**, green), were immobilised o/n on treated glass surface, were stained with Alexa647-anti-human-IgG (αhuman-IgG-Alexa647) (**H**, red). Nanocarrier structures show prominent staining of αhIgG-Alexa647 of the targeting cetuximab antibodies only on surface regions and siRNA within the vesicles (**I**, overlay).

The functional relevance of the unbound excess of SMCC-protamine as well as the presence of the targeting antibody to bind, carry and internalise siRNA into target cells necessitates a complex carrier structure. We therefore tested the reaction mixtures in cell-free environments for the presence of particles of a size that cannot be explained by a linear composition of simply one mAB-SMCC-protamine conjugate binding several siRNAs. Indeed, in dynamic light scattering (DLS) spectroscopy, a size-motion correlation that is based on Brownian molecular motion [11], the single components such as the antibody-conjugate-protamine without siRNA and the purified and depleted counterpart and the SMCC-protamine alone showed DLS values related to totally plausible sizes of 10–20 nm (Fig. 3A, blue and red curves), representing the monomeric conjugate. In contrast, the non-purified αEGFR-protamine conjugate, when combined with siRNA led to the existence of a large particle 427 ± 12 nm in size (Fig. 3A, black curve). Again, this observation was not made when SMCC-protamine was depleted (Fig. 3A, red curve). This particle formation occurred spontaneously. The zeta-potential was assessed with the zeta-counter −5.7 ± 3.2 mV, so in a fairly anionic range tending to electroneutrality. In the end, the structures were large enough to be seen in fluorescence micrographs: in cell-free environment, after overnight incubation and mounting on adhesive slides, αEGFR-protamine (αEGFR-mAB-P) preparations still containing free SMCC-protamine (αEGFR-mAB-P/P) with siRNA showed particles representing the size range seen in the DLS measurements (Fig. 3B, C). The structures were of spheroid, micellar form and light breaking enough to be seen even in bright field microscopy (Fig. 3D). The size of those vesicular structures varied between 0.5 and 2 µm and, more importantly, was microscopically stable for many hours even in unprotected environment. When the conjugates were depleted from free SMCC-protamine, these structures were no longer detectable (Fig. 3E).

αEGFR-protamine/P-siRNA nanocarriers were next subjected to electron microscopy, revealing spheroid particles also of 100–200 nm size after 2 h of auto-assembly (Fig. 3F). In order to shed light on the fine-structure of the nanocarriers, we attached αEGFR-protamine/P-Alexa488-control-siRNA nanocarriers to treated glass surfaces and performed immunostaining for the human-IgG proportion, which stains only a faint rim section of each nanocarrier structure (Fig. 3H). We explain this with the αEGFR-IgG facing outward, while the majority of Alexa488-siRNA signals fill the lumen of the particle (Fig. 3G).

**Fig. 4:**
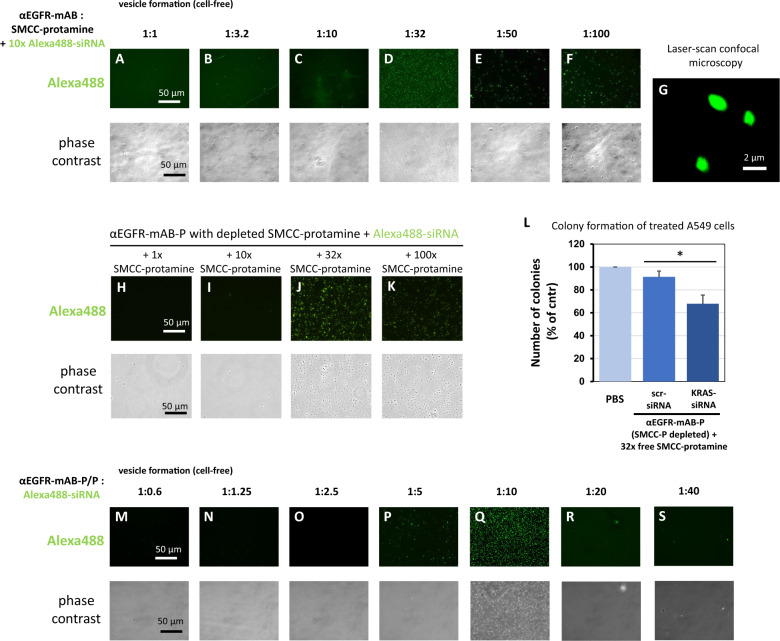
Deciphering preconditions for effective nanoparticle formation between anti-EGFR-mAB-SMCC-protamine conjugate, free SMCC-protamine and siRNA. **A**–**F** αEGFR-mAB was conjugated with rising excess of free SMCC-protamine ranging from 1:1 molar ratio to 1:100 excess of SMCC-protamine in chamber slides (see Fig. 1A for reaction details). Resulting conjugates were used to bind siRNA in a cell-free standardised assay. The 1:32 ratio mAB to SMCC-protamine formed a homogeneous population of stable particles in the range up to 0.5–2 µm (**D**), see detail in **G**, whereas the other conjugates were incompetent to form stable particles. Stable particles subjected to confocal laser scan (CLS) microscopy optical sections showed a homogeneous distribution of fluorescent Alexa488 signals within the particle (**G**). **H**–**K** αEGFR-mAB-P depleted of free SMCC-protamine can form nanoparticles when at 32x free SMCC-protamine (**J**) is re-added to the conjugate with Alexa488-siRNA. **L** αEGFR-mAB-P depleted of free SMCC-protamine is effective in the inhibition of A549 cell colony formation when 32x free SMCC-protamine is re-added to the conjugate with anti-KRAS-siRNA in contrast to unspecific control (scrambled, scr) siRNA. Mean ± SD of three independent experiments. Two-sided *t*-test, *p < 0.05 (two-sided *t*-test). **M**–**S** Vesicle formation with 60 nM αEGFR-mAB-P in presence of 32x SMCC-protamine and rising (1:0.6–1:40) molar ratios of Alexa488-control-siRNAs compared to the antibody concentration. Vesicle formation can be observed at 5 to 10x molar excess of siRNA (**P**, **Q**). Upper panels: Fluorescence microscopy of Alexa488-siRNA positive vesicle. Lower panels: Phase contrast of the same preparations as in upper panels.

Next, we further analysed the properties of the different conjugation products according to their capacity to form vesicular structures without cells when these conjugation products were incubated with Alexa488-control-siRNA (Fig. 4A–F). When the αEGFR-mAB-SMCC-protamine (αEGFR-mAB-P) was conjugated with free SMCC-protamine at ratios of 1:1 up to 1:10, no efficient cell-free vesicle formation could be observed (Fig. 4A–C). At the ratio of 1:32, the vesicle formation was abundant (Fig. 4D) and it decreased again at higher ratios of 1:50 and 1:100 (Fig. 4E, F), which corresponded with the cellular internalisation capacity of these complexes (Fig. 1I–N) and efficiency in targeting KRAS in colony assays (Fig. 1O–T). The micellar structure appeared round and completely filled with Alexa488-siRNA also in CLS microscopy (LSM; Fig. 4G). This suggests an optimal nanoparticle formation of αEGFR-mAB-P to free SMCC-protamine ratio of 1:32, corresponding to the efficient siRNA delivery and internalisation into cells. Once formed properly by auto-assembly, the nanocarriers demonstrated a high stability towards pH shifts between pH 4.8 and 8.0 (Supplementary Fig. 3A) as well as tolerance towards exposure to serum between 10 and 50% (Supplementary Fig. 3B), both are important factors for a safe applicability in vitro and in vivo.

To further elucidate the function of SMCC-protamine coupled to the antibody and as free molecule within the complex, we re-added and titrated the amount of free SMCC-protamine to the antibody-protamine-conjugates that were previously depleted from free SMCC-protamine by HPLC as described in Fig. 2. The antibody-protamine conjugates without free SMCC-protamine are not able to form vesicular structures with fluorescent siRNA as depicted in Fig. 3F. We therefore addressed whether free SMCC-protamine can be re-added to fulfil this electrostatic connector-function and if SMCC-protamine can be substituted by protamine without sulfo-SMCC. We added different amounts of free SMCC-protamine and protamine alone to αEGFR-mAB-P and Alexa488-siRNA (Fig. 4H–K). The addition of 1x SMCC-protamine or 10x SMCC-protamine in relation to the antibody were not effective to form vesicular structures (Fig. 4H, I), while the addition of 32x SMCC-protamine lead to a very effective formation of vesicles (Fig. 4J) and the addition of 100x SMCC-protamine to some vesicle formation (Fig. 4K). Moreover, the functional assay revealed that αEGFR-protamine first depleted from SMCC-protamine and later re-complemented with 32x SMCC-protamine complexed with anti-KRAS-siRNA significantly inhibited colony formation of A549 cells when compared to untreated or control scr- (scrambled) siRNA treated cells (Fig. 4L).

Finally, we asked if siRNA is needed to form vesicles. We incubated a constant amount of αEGFR-mAB-P with constant 32x free SMCC-protamine with different amounts of Alexa488-control-siRNA (Fig. 4M–S, green fluorescence in upper panels, phase contrast in lower panels). Remarkably, nanoparticles are only efficiently formed with an optimal molar excess of siRNA of 5–10 times over the antibody (Fig. 4P, Q).

Thus, the three components (1) antibody-SMCC-protamine, (2) siRNA and (3) unbound SMCC-protamine form a stable nanostructure necessary for functional activity of the nanocarrier.

### αEGFR-mAB-protamine/KRAS-siRNA/protamine nanocarriers inhibit growth of EGFR-positive NSCLC cells in vitro and in vivo

**Fig. 5:**
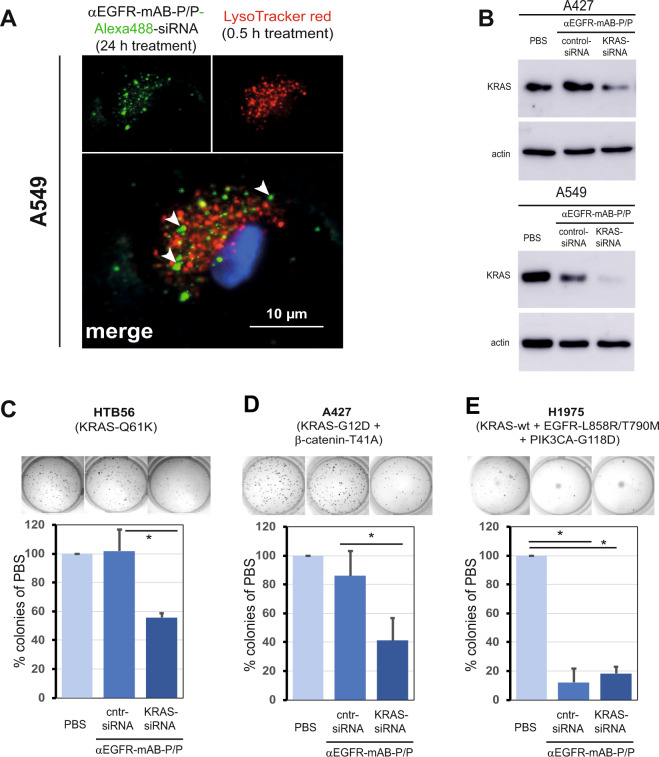
The protamine-conjugated EGFR-monoclonal antibody complex binds siRNA, internalises via the endosomal pathway specifically into EGFR-expressing cells, inhibits KRAS expression upon KRAS-siRNA transport, and specifically inhibits cellular colony formation. **A** Vesicular tracking in A549 NSCLC cells. Cells treated with αEGFR-mAB-P/Alexa488-siRNA/P were subjected to Lysotracker red staining. The vesicles containing Alexa488 rarely colocalized with Lysotracker staining (merged picture see white arrows for green Alexa-siRNA fluorescence outside red lysosomes). **B** Western blot for KRAS of two NSCLC cell lines treated with PBS, carrier-control-siRNA and carrier-KRAS siRNA accompanied by blot for actin as loading control (same blot). **C**–**E** In vitro response of colony growth to treatment with KRAS mutation-specific siRNAs. Cell lines indicated were pre-incubated with antibody-conjugates coupled to siRNAs, resuspended in soft agar and cultivated for ~1 week. Cntr-siRNA, control-siRNA; PBS, vehicle control without active treatment. Mean ± SD of three independent experiments. Two-sided *t*-test, *p < 0.05. Specific molecular characteristics of the cell lines are given in parenthesis.

As a first example for the efficiency of our modular nanoparticle therapy platform, we applied αEGFR-mAB-P/siRNA/P nanoparticles to highly EGFR-positive NSCLC cells. These nanoparticles internalised into five EGFR-positive NSCLC cell lines with high efficiency (Supplementary Fig. 6). Furthermore, we found receptor-mediated Alexa488-siRNA rarely in lysosomes (Fig. 5A, stained in red by LysoTracker), but distinct from those in non-acidic cellular compartments representing early endosomes.

In order to silence the important oncogenic expression of KRAS in NSCLC cells, we used siRNAs targeting wild type KRAS. EGFR-antibody-nanostructures carrying KRAS-siRNA, but not control-siRNA treated cells showed marked reduction of KRAS protein expression levels in EGFR-positive NSCLC cell lines A549 and A427 as determined by Western blot analysis (Fig. 5B).

Next, in addition to what is shown for NSCLC A549 cells in Fig. 2G and for SK-LU1 cells in Fig. 2H, additional KRAS-mutated NSCLC cell lines, namely HTB56 (Fig. 5C) and A427 (Fig. 5D), with the mutation patterns defined in the Fig. 5 and accomplished KRAS-siRNA-knockdown (Fig. 5B), also showed significantly decreased colony growth (Fig. 5C, D) when treated with appropriate KRAS-siRNA nanocarriers as compared to cells treated with control-siRNA carriers or untreated cells (phosphate buffered saline (PBS)). In contrast, the lung cancer cells line harbouring an EGFR-mutation, but not a KRAS mutation, H1975 (KRAS-wild type, Fig. 5E) does not show inhibited colony growth by specific KRAS-inhibition. H1975 cells are very sensitive to the αEGFR-antibody-protamine complex, an effect that was reported before with unconjugated antibody [12]. Here, H1975 cells showed inhibited colony growth also treated with αEGFR-nanocarriers coupled to control-siRNA without a further growth inhibition by KRAS knockdown (Fig. 5E). These observations underline the functional specificity of our siRNA loaded nanostructures.

**Fig. 6:**
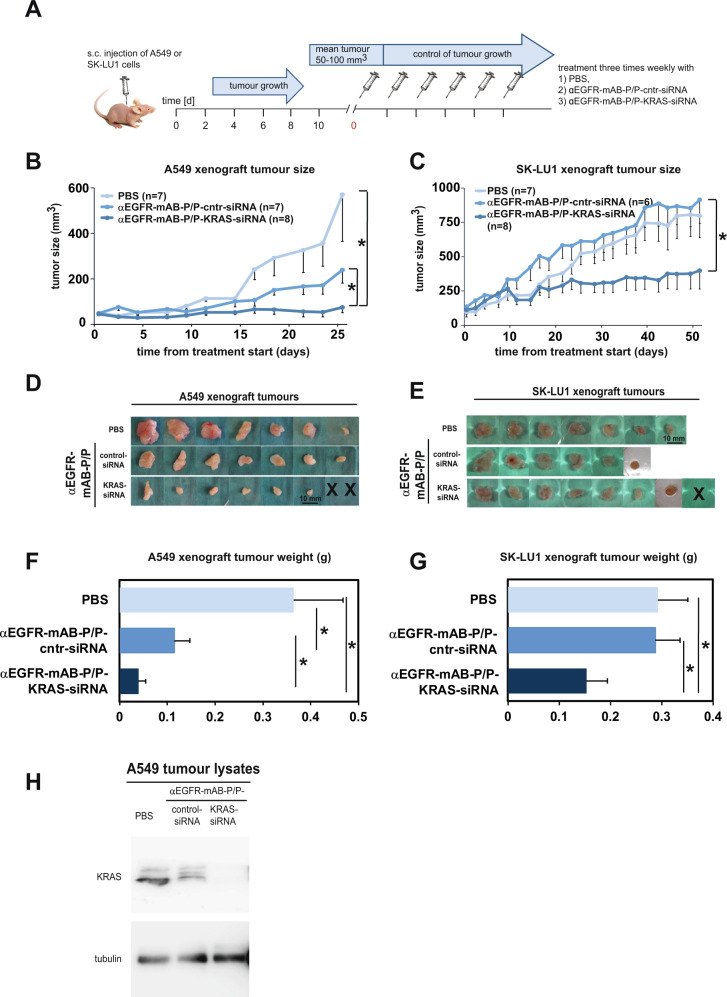
NSCLC xenograft growth is decreased upon knockdown of KRAS via the anti-EGFR-mAB-protamine (P)/siRNA/P nanostructures. **A** Treatment scheme of the in vivo experiments. **B**–**F** Results of systemic in vivo application of targeted nanocarriers on A549 and SK-LU1 xenograft tumours. **B** Tumour growth curves for A549 (means–SEM). **C** Tumour growth curves for SK-LU1 (means–SEM). Both tumour growth curves show significant reduction of tumour growth in anti-KRAS siRNA treatment groups. **D**, **E** Size of excised tumours at the end of the respective experiments (X = individual tumours in anti-KRAS siRNA group were treated to extinction). **F**, **G** Weight statistics of the excised tumours (g, gram). **H** Proof of KRAS oncogene knockdown in excised tumour tissue by KRAS western blot ex vivo. Cntr control scrambled siRNA. *denote significant differences between groups (*p* < 0.05, two-sided *t*-test).

To investigate the antitumour activity of αEGFR-antibody-P/KRAS-siRNA/P nanostructures in vivo, we xenotransplanted human A549 and SK-LU1 cells, respectively, subcutaneously in the flanks of CD1-nude mice and treated them systemically by intraperitoneal (i.p.) route as depicted schematically in Fig. 6A. In the A549 xenografted mice, the treatment with αEGFR-mAB-P/KRAS-siRNA/P nanostructures led to significantly reduced tumour growth compared to control-siRNA loaded αEGFR-mAB-P/P nanostructures (Fig. 6B). The SK-LU1 tumour growth was significantly inhibited when mice were treated with αEGFR-mAB-P/KRAS-siRNA/P nanostructures compared to PBS control group and to αEGFR-mAB-P/control-siRNA/P nanostructures (Fig. 6C).

After 26 days of treatment the A549 tumours were dissected and weighed (Fig. 6D, F). The A459 tumour weights of the group treated with αEGFR-mAB-P/KRAS-siRNA/P nanostructures were significantly diminished in comparison to the control-siRNA loaded αEGFR-mAB-P/P nanostructure group (Fig. 6F). The sizes of isolated anti-KRAS-treated xenografts were smaller (Fig. 6B, D). Also, in SK-LU1 xenografted mice, the application of the αEGFR-mAB-P/KRAS-siRNA/P nanostructures reduced tumour sizes and weights by about 50% when compared with the control-treated groups (Fig. 6C, E, G). Western blot analysis revealed KRAS knockdown in xenograft tumours after αEGFR-mAB-P/KRAS-siRNA/P nanostructure treatment (Fig. 6H). We conclude that the αEGFR-mAB-P/KRAS-siRNA/P nanostructures reach the tumour, internalise into accessible cells and release the siRNA into the cytoplasm of tumour cells to induce the KRAS knockdown. The dependence of KRAS-mutated NSCLC-tumour cells then led to decreased proliferation due to the lack of KRAS protein.

Tumours treated with αEGFR-mAB-P/KRAS-siRNA/P nanostructures had reduced proliferation rates compared to PBS or control-treated tumours as determined by detection of the proliferation marker Ki67 by immunofluorescence (Supplementary Fig. 7) and higher apoptosis rates as determined by TUNEL staining (Supplementary Fig. 7). Accordingly, the marked and significant reduction of the tumour sizes by treatment with the αEGFR-mAB-P/KRAS-siRNA/P nanostructures can be explained by a combination of reduced proliferation and increased apoptosis in the respective tumours.

To test for safety and tolerability repeated controls of the body weight of the animals were performed and showed no differences between the single therapy groups (Supplementary Fig. 8A, B).

In summary, systemic treatment of NSCLC xenografts in mice with αEGFR-mAB-protamine-KRAS-siRNA-free SMCC-protamine nanostructures was successful, tumour-specific and tolerable.

### αIGF1R-mAB-P/EWS-FLI1-siRNA/P nanoparticles significantly inhibit growth of IGF1R-positive Ewing sarcoma cells in vitro and in vivo

To test the therapeutic siRNA nanocarrier platform for general applicability, we extended our experiments to Ewing sarcomas. Ewing sarcomas are mesenchymal malignant tumours in children and young adults, with poor outcomes especially for metastatic tumours and after relapse [13]. The key genetic event in Ewing sarcoma is a chromosomal translocation t(11;22) that results in the formation of the fusion protein EWS-FLI1 which acts as driver of tumour growth [14]. Ewing sarcoma cells express high amounts of IGF1R on their surface. We therefore intended to apply our modular therapy using anti-IGF1R-antibodies such as the clone ImcA12 (cixutumumab) [15] or RG-1507 (teprotumumab) [16] as SMCC-protamine conjugates to transport EWS-FLI1 breakpoint-specific siRNA. To this end, we used the EWS-FLI1 positive SK-N-MC cell line, which originally was thought to be of neuroblastoma origin, but later was recategorized as originating from an Ewing family sarcoma and which is highly IGF1R-positive. Preliminary results targeting the IGF1R receptor in another Ewing cell line were published by us before [6].

**Fig. 7:**
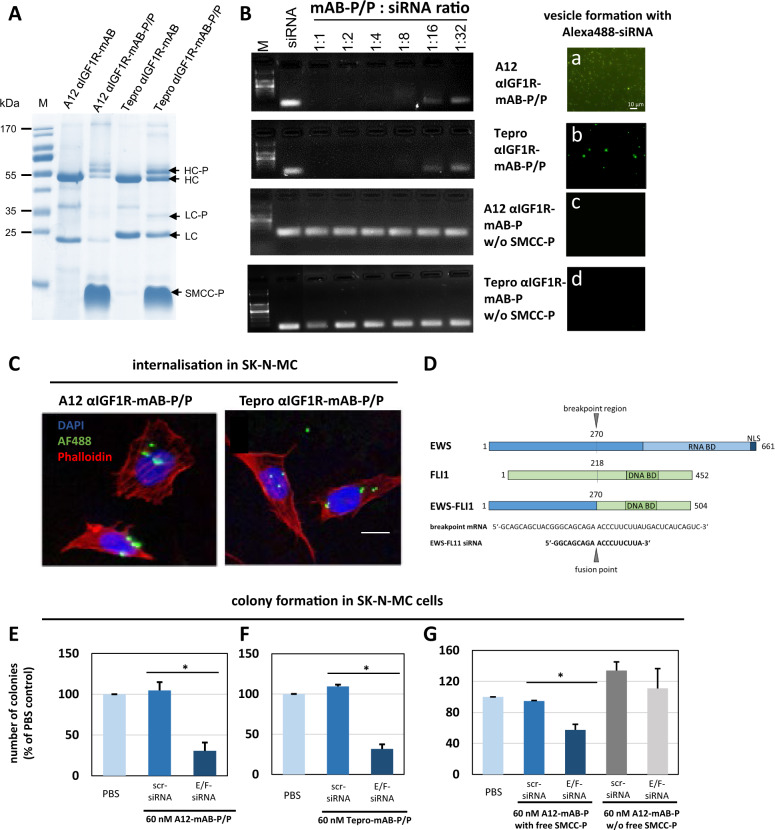
Knockdown of Ewing-specific EWS-FLI1 fusion protein by αIGF1R-mAB-protamine/siRNA/protamine nanoparticles targeting Ewing sarcoma cells. **A** IGF1R-targeting mABs A12 (cixutumumab) and Tepro (teprotumumab) were expressed and purified using a GMP-like method and then conjugated to protamine to enable siRNA binding and transport. IgG-protamine conjugates exhibit a decent molecular weight shift (arrows). HC heavy chain, LC light chain, -P SMCC-protamine. **B** Band-shift assay using αIGF1R-mABs-protamine (-P) and different ratios of siRNA (left panels). Antibody-protamine complexes without free SMCC-protamine do not bind siRNA anymore. Right panels (a–d): αIGF1R-mAB-P in presence of free SMCC-protamine can form nanoparticles with Alexa488-control-siRNA (a: A12, b: Tepro). αIGF1R-mAB-P depleted from free SMCC-protamine cannot form these nanoparticles with Alexa488-control-siRNA (c: A12, d: Tepro). **C** Anti-IGF1R-mAB-protamine with free SMCC-protamine shuttled Alexa488-marked control-siRNA to SK-N-MC Ewing cells (left panel: A12-mAB-P, right panel: Tepro-mAB-P). Bar represents 10 µm. **D** Schematic representation of the breakpoint regions in the human EWS protein, the FLI-1 protein and the resulting oncogenic fusion protein EWS-FLI1 along with the breakpoint mRNA sequence and the EWS-FLI1-specific siRNA sequence that was used for this study (see **E**–**G** and Fig. 8) (BD binding domain, NLS nuclear localisation signal). **E**–**G** SK-N-MC cells were treated with protamine-conjugated A12 (**E**) or Tepro (**F**) formed siRNA nanoparticles as indicated and subjected to colony formation assays. E/F–siRNA is an siRNA interfering with the mRNA of the driving Ewing sarcoma EWS-FLI1 as depicted in **D**. **G** SK-N-MC cells treated with αIGF1R (A12)-mAB-protamine/EWS-FLI1-siRNA/P nanoparticles form significantly less colonies in soft agar than cells treated with αIGF1R (A12)-mAB-protamine/contr (scr)-siRNA/P. No differences in colony formation can be observed when SK-N-MC cells were treated with αIGF1R-mAB-protamine conjugates without free SMCC-protamine not resulting in the formation of nanocarriers. Shown here are means of three independent experiments ± SD. Asterisk indicates significant differences (*p* < 0.05, two-sided *t*-test).

**Fig. 8:**
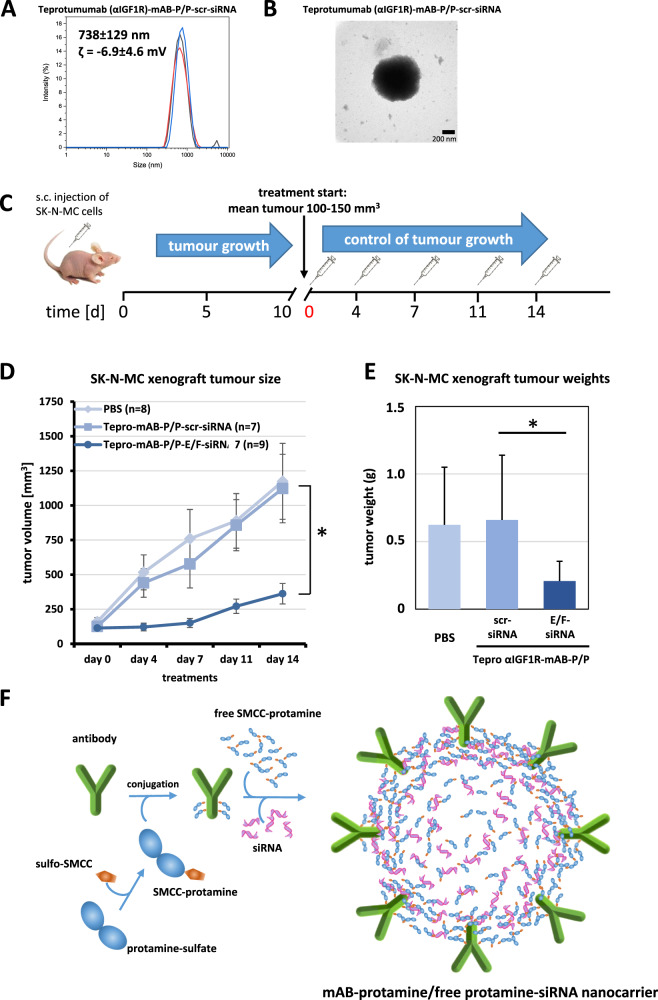
Ewing sarcoma xenograft tumour growth is inhibited upon knockdown of oncogenic EWS-FLI1 translocation product through systemic therapy with αIGF1R-mAB-protamine-siRNA-protamine nanocarriers. **A** Dynamic light scattering spectroscopy (DLS) of teprotumumab (αIGF1R)-mAB-protamine-siRNA-protamine nanocarriers reveals a particles size of 738 ± 129 nm with a zeta-potential of −6.9 ± 4.6 mV. **B** αIGF1R-mAB-P/P-scrm siRNA nanoparticles were left to form for 2 h and subjected to electron microscopy on copper grids by phospho-Wolfram negative staining. **C** Treatment scheme of the in vivo experiments. Nanoparticles were given intraperitoneally as visualised. **D**, **E** Results of systemic in vivo application of targeted nanocarriers on SK-N-MC xenograft tumours. **D** Tumour growth curves SK-N-MC treated with αIGF1R-mAB teprotumumab (“Tepro”)-protamine-siRNA/P nanoparticles (means ± SEM; two-sided *t*-test, *p < 0.05). **E** Weight statistics of the excised tumours at the end of the experiment (mean ± SD. Two-sided *t*-test, *p < 0.05). **F** Illustration of a cross section through an idealised nanoparticle structure fulfilling the conditions for an effective antibody-protamine-siRNA-SMCC-protamine nanocarrier complex deduced from our experiments. Electrostatic binding bridges are formed between mAB, with some protamines (cationic) coupled to the targeting antibody, siRNA (anionic), and free SMCC-protamine (cationic). The nanostructures assemble spontaneously into the optimal and most stable electrostatic status and function as nanocarriers for siRNA.

We cloned and expressed the two different IGF1R-antibodies in CHO-S cells and purified them using HPLC. Successful clones were obtained using sequences from cixutumumab (here referred to as “A12”) and teprotumumab (here referred to as “Tepro”), of which the latter recently has been approved for thyroid eye disease [16]. Both antibodies were produced in sufficient amounts and coupled to SMCC-protamine (Fig. 7A), both bind siRNA (Fig. 7B, left panels) and form nanoparticles only in presence of free SMCC-protamine, just as seen for the αEGFR-mAB-P (Fig. 7B, right panels: a–d). Both αIGF1R-mAB-P/P nanoparticles transport fluorescent siRNA into IGF1R-positive cells SK-N-MC (Fig. 7C). Next, we used a previously published siRNA sequence [17] that was successfully used to knockdown the Ewing-specific EWS-FLI1 fusion protein by targeting its mRNA spanning across the fusion point (Fig. 7D). When EWS-FLI1-positive SK-N-MC cells were incubated with these αIGF1R-mAB-protamine conjugates in complex with siRNA against EWS-FLI1 and seeded in semisolid soft agar, colony formation was significantly reduced (Fig. 7E, F). This was dependent on the presence of free SMCC-protamine and nanocarrier formation, while independent of a possible inhibitory effect of unconjugated αIGF1R-mAB (Fig. 7G and Supplementary Fig. 4B). Further characterisation of teprotumumab αIGF1R-mAB-P/P-siRNA nanoparticles reveal a mean size of around 738 nm as determined by DLS (Fig. 8A), which was confirmed by electron microscopy (Fig. 8B). We concluded that both αIGF1R-mAB-protamine nanoparticles transported sufficient amounts of anti-EWS-FLI1-siRNA into these cells to inhibit their growth, an effect that we also wanted to analyse in vivo.

To this end, we xenotransplanted 10^7^ human SK-N-MC cells subcutaneously (s.c.) into the flank of CD1-nude mice and treated cohorts of at least 7 mice with either PBS or αIGF1R-mAB-P/P in complex with scrambled control-siRNA, or in complex with the above mentioned EWS-FLI1-siRNA i.p. (Fig. 8C, D). Treatment was started when tumours had reached an average size of 100-150 mm^3^. Tumours in the treatment group that obtained Tepro-mAB-P/EWS-FLI1-siRNA/P nanoparticles showed a significant and almost complete growth inhibition when compared to both control groups (Fig. 8D, E). This suggested that the knockdown of EWS-FLI1 via Tepro-mAB-P/siRNA/P nanoparticles was successful after systemic in vivo application, with no toxicity detected according to the mouse weight (Supplementary Fig. 8C).

## Discussion

In mammals, in the course of spermiogenesis, the packaging of DNA is changed from all somatic chromatin to one in which the complete DNA is tightly coiled by protamines into toroidal structures containing up to 60 Kb of base sequence and this process that can be reproduced in vitro [18]. DNA molecules packaged by protamine have also been shown to be more resistant to degradation by nucleases by reaching densities close to crystallised DNA. Consequently, 60 Kb of DNA containing toroids are only 100 nm in size in order to pack the complete genomic DNA into a small and hydrodynamic sperm head.

In this study, we unexpectedly observed the formation of a protamine-induced structure totally different from the DNA packaging by the coordination of short 21 base pair double stranded siRNAs: The assembly is started with a chemical conjugation of the targeting antibody with protamine including subsequent purifications steps, followed by an electrostatic self-assembly between the anionic cargo siRNA and both cationic coordinators, SMCC-protamine and antibody-protamine (Fig. 8F). Here, the resulting structure is much less condensed and spontaneously forms nanoparticles in the size range of ~100–200 nm after 2 h, which must account for another form of nucleic acid-protamine interaction than seen with longer DNA molecules. These nanostructures function as nanocarriers for siRNA and allow for a platform technology with modular changes for specificity of the targeting antibodies and the siRNA.

To our knowledge, such an electrostatic micelle-like structure was never observed for a targeted protamine-containing nanoparticle before. This is the first time that a nanoparticle is structurally described and efficiently used for therapy that is formed by an antibody-SMCC-protamine molecule, free SMCC-protamine and a negative component such as siRNA (α-P/siRNA/P).

Protamine has been intensively studied as a carrier- and coordination molecule for substances other than DNA, for instance solid lipid nanoparticles [19], gold nanoparticles [20] and the saccharide heparin [1] and of course, for the complexation of siRNA [21]. Heparin is an aminosaccharide of 15 kDa median size, an anticoagulant poly-anionic substance. Interaction studies of heparin and protamine revealed the formation of protamine-heparin aggregates [3]. Given the fact that the median molecular weight of heparin and siRNA is similar, as well as the basic structure of aminosaccharide backbone connected to anionic sulfate group instead of phosphate in the RNA, one can assume a comparable coordination forming the nanoparticles between the anionic components and protamine-antibody conjugates. Interestingly, the particles formed by only SMCC-protamine and siRNA were much smaller than those formed by those components with the added protamine-conjugated antibody (6 nm vs. 100 nm) and were of a more variable size. Together with the observation that the targeting antibody is necessary to make a contact of the nanoparticles to the intended target cell, we deduce that the antibodies are exposed on the outside of the micellar structure and may act also as a scaffold to keep the spheroid structure intact (Fig. 8F). Such an outside position of the targeting antibody could indeed be shown by immunofluorescence studies of the carriers against IgG (Fig. 3I). By titration of the molar ratios of the components to each other, a ratio of 1 mol of targeting antibody-protamine to 32 molar excess of free protamine complexing 5–10 mol of siRNA was found to build the most stable nanoparticles, which in addition were very efficient as nanocarriers for the treatment of cancer cells when relevant antibodies are used for targeting and relevant oncogene-interfering siRNAs are chosen. Because of the methodology used, the therapeutic results previously published by us using antibody-protamine-siRNA constructs for the treatment of colorectal tumours [6–8] must be reinterpreted to be based on these nanostructures described in this paper. Here, we show that the application of these siRNA loaded nanostructures is modular and quite easily applicable against cancer types as diverse as NSCLC and Ewing sarcoma. Both cancers in an advanced stage are still disastrous in their outcome, despite some progress concerning molecular targeted therapies [22]. In the treatment of NSCLC, inhibitors of several driver molecules and pathways are available and in clinical use. Unfortunately, only subgroups of patients benefit from small molecule-inhibitor-based targeted therapy and resistance develops almost universally during treatment [23]. In Ewing sarcoma, targeted therapy has not changed the outcome of the patients up to now, and our observation that systemic therapy with EWS-FLI1 siRNA nanocarriers can effectively inhibit Ewing sarcoma xenograft growth has to be further studied for its potential of translation into the clinic.

## Materials and methods

### siRNA-carrier construction

For expression of anti-IGF1R-antibodies, published sequences of the heavy chain and light of cixutumumab (“A12”[15]) and teprotumumab (“Tepro”[16]) were cloned into the bicistronic expression vector pVITRO-neo, preceded by a rituximab leader sequence and followed by an IRES-GFP cassette. Plasmids are available on request. The resulting plasmids were transiently transfected into CHO-S cells. After clonal expansion of flow cytometrically sorted GFP-positive single cells, best antibody-expressing clones were identified by dot blot analysis of the supernatants. These clones were cultured in 300 ml power-CHO medium (Biozym, Hessisch Oldendorf, Germany) at 31 °C for 6 days and supernatants were subjected to HPLC purification using a protein G-sepharose matrix (teprotumumab) or a CH1 matrix (cixutumumab) via an ÄKTA pure chromatography system (GE General Electric, MA, USA). Fractions were eluted with glycine buffer (100 mM glycine-HCL, pH 2.5) and subjected to dialysis in PBS overnight (o/n) at 4 °C.

The conjugation methodology has been described by us before [7]. In this study, we have used the following modifications as described in brief. Protamine sulfate (3 mM) was amino-terminally coupled to the bifunctional crosslinker sulfo-SMCC (no. 13415, CovaChem, Loves Park, IL, USA) in a 1:5 molar ratio in amino-free PBS buffer. The pH was adjusted to pH 7.0 with 0.1 M carbonate buffer (pH 8.3). The mixture was left to react for 1 h at room temperature (RT), protamine doublets were separated by gel filtration chromatography in Zeba spin desalting columns (No. 89891, Thermo Fisher Scientific, Waltham, MA, USA), and then protamine-sulfo-SMCC (for brevity designed as “protamine” or “-P” throughout the paper) was coupled to cysteine residues of antibody cetuximab (31 µM stock; Erbitux^TM^, Merck-Serono, Darmstadt, Germany) or the anti-(α)IGF1R-antibodies A12 and Tepro produced in our laboratory under GMP-like conditions as described above in a 32:1 molar ratio or as indicated in Fig. 1A at 4 °C o/n. The resulting antibody-protamine adducts were stored at 4 °C and were stable for several weeks. siRNA duplexes were bound to antibody-protamine in a 4 to 10-fold molar excess at RT for 1–2 h. This complex was prepared freshly before use.

After chemical conjugation, αEGFR-mAB-protamine containing unbound SMCC-protamine in molecular excess is applied to protein G-sepharose equilibrated with PBS, washed with 10 CV of PBS and then eluted with a steep gradient of 100 mM glycine-HCL pH 2.5. Fractions were collected and checked for presence of unbound SMCC-protamine by SDS-PAGE and Coomassie stain. Fractions depleted of unbound SMCC-protamine were subjected to further analysis.

For the estimation of siRNA coupling, stability and internalisation efficiency, antibody-protamine conjugates were coupled to scrambled siRNA (see below), Allstars negative control-siRNA-Alexa488 (cat. no. 1027284, Qiagen, Hilden, Germany) or Allstars negative control-siRNA-Alexa 555 (cat. no. 1027286, Qiagen). Treatment experiments were done using siRNA duplexes against KRAS (KRAS-siRNA sense: 5′-UUC UGC UUG UGA CAU UAA AAA, EWS-FLI1-siRNA: 5′-GGC AGC AGA ACC CUU CUU AUU-3′) and as control a scrambled siRNA (sense: 5′ GGC CGA CAC CGU CAU UUA ATT (all from Microsynth, Balgach, Switzerland). Band-shift assays were performed as described previously [7].

### Cell culture

The EGFR-positive NSCLC cell lines A427, A549, HTB56, SK-LU1 and H1975 were treated with antibody-siRNA-complexes as described previously [7, 8]. The cell lines A549 and SK-LU1 were cultivated in DMEM, A427, H1975, SK-N-MC and CHO-S in RPMI, all supplemented with 10% foetal bovine serum (FBS) and 1% penicillin and streptomycin (PS). HTB56 was cultivated in MEM, supplemented with 20% FBS, 1% PS, 1% sodium pyruvate and 1% non-essential amino acids. CHO-S cells were shifted to FCS-free power-CHO medium and 31 °C shaking in glass bottles for expression of antibodies. Cell lines were obtained from ATCC (Manassas, VA, USA) or CLS (Eppelheim, Germany) and routinely tested for identity and mycoplasma absence.

### Fluorescence microscopy

Cell lines were seeded at 2 × 10^4^ cells/cm^2^, cultivated on sterile cover slips o/n and treated with 60 nM antibody-protamine or PBS incubated with Alexa Fluor 488-labelled Allstars negative control-siRNA (Qiagen 1027284) and Allstars negative control-siRNA-Alexa 555 (Qiagen 1027286) at 1:10 molar ratio each o/n at 37 °C and 5% CO_2_. Subsequently, cells were washed with PBS, fixed with ice-cold 4% paraformaldehyde (PFA), stained with Hoechst 33342, mounted with Dako fluorescence mounting medium and photographed on a Zeiss Axioskop. To detect endosomal escape, cells that internalised cetuximab-Alexa488-siRNA overnight were incubated with 60 nM LysoTracker^TM^ red DND-99 (cat. No. L7528, Invitrogen, Carlsbad, CA, USA) for 30 min, then washed, fixed, stained with Hoechst and mounted with Dako fluorescence mounting medium and photographed as described above.

For cell-free nanoparticle-self-assembly studies, the antibody-P Alexa488-siRNA conjugates were applied to cell-culture treated chamber slides overnight in order to settle the nanoparticles by gravity, washed with PBS, fixed with 4% PFA, stained with Alexa647-anti-hIgG (Dianova, Germany, #109-607-003) and mounted in DAKO mounting medium for microscopical analysis.

### Electron microscopy

Freshly prepared nanoparticles were sedimented on an formvar-coated, carbon-sputtered copper grid. After negative staining with 1% phosphor-tungstic acid, pH 7, the samples were analysed at 80 kV on a Tecnai 12 electron microscope (Fei, Eindhoven, The Netherlands). Images of selected areas were documented with Veleta 4k CCD camera (Emsis, Münster, Germany).

### Flow cytometrical analysis

For flow cytometrical analysis, extracellular fluorescence was quenched using trypan blue to a final concentration of 0.2%, which does not enter intact cells [10]. The intracellular fluorescence intensity of Alexa488 (Channel 525/40 BP) was measured by flow cytometry using the CytoFLEX S Flow Cytometer (Beckman Coulter, Brea, CA, USA) in triplicates (see Supplementary Fig. 6 for examples). The data were analysed and expressed as geometric means of fluorescence of 1000 events per triplicate.

### Confocal laser scan microscopy

For confocal laser scan (CLS) microscopy, A549 cells were grown on 10 mm glass coverslips and treated with the coupled αEGFR-mAB-P-siRNA/P nanostructures to a final concentration of 600 nM (siRNA). The cells were washed three times with PBS, fixed using 4% (w/v) PFA in PBS (20 min), quenched with 0.2% (w/v) glycine in PBS (20 min) and permeabilized using 0.2% (w/v) triton X-100 in PBS for 10 min). Actin and DNA were stained with phalloidin-tetramethyl rhodamine isothiocyanate (TRITC; 1:500 in PBS, 1 h) and with Draq5 (BioStatus, Shepshed, United Kingdom; 1:500 in PBS, 20 min), respectively. After further washing, the preparations were embedded in fluorescence mounting medium (Dako/Biozol, Eching, Germany) and analysed with a Zeiss LSM 510 Meta CLS microscope equipped with a Plan-Apochromat 63/1.4-numeric-aperture oil immersion objective (Carl Zeiss, Oberkochen, Germany).

### Dynamic light scattering (DLS)

Particle size detection by means of DLS was performed on a zeta-counter (MALVERN, Malvern, United Kingdom), which correlates light diffusion caused by particles in a solution to their size and the zeta-potential. Measurements were performed in at least three technical replicates.

### Western blots

In total, 5 × 10^5^ cells of each cell line were seeded and cultivated overnight, treated with cetuximab-protamine (60 nM) coupled to the indicated siRNAs at 1:10 molar ratio once a day for 3 days, harvested, lysed in RIPA buffer and cleared by ultrasonification and centrifugation. Xenograft tumours were homogenised as 10% w/v in RIPA buffer using an ultraturrax, cleared by ultrasonification and centrifugation. Western blot analysis was performed using standard protocols with the following antibodies: anti-KRAS (ab55391, Abcam, Cambridge, UK), and anti β-Actin antibody (Clone AC-15, Sigma Aldrich).

### Clonogenic tumour-cell growth in soft agar

In brief, 4000 trypsinised cells in 120 µl per sample were incubated with antibody-protamine coupled in a 1:10 molar ratio to the indicated siRNAs with free SMCCC-protamine upon ~2 h of spontaneous nanobody assembly at 60 nM end concentration for 1 h at RT, resuspended in 168 µl of 3% agar (Difco Agar Noble) and 432 µl adequate cell-culture medium and cultivated in triplicate for colony formation in 96-well format (180 µl/well). After one week, the assays were stained with 20 µl 4 mg/ml Iodonitrotetrazolium chloride solution and incubated over night at 37 °C. The next day, the assays were counted for colony numbers.

### Mouse xenograft tumour model

All animal experiments in this study were carried out in accordance with the recommendations of the Institutional Animal Care and Use Committee “Landesamt fuer Natur, Umwelt und Verbraucherschutz NRW” (LANUV). This study was performed with permission of the Institutional Animal Care and Use Committee and of the local veterinary administration of Muenster (Permit no. 84-02.04.2015.A158 and 81-02.04.2020.A001). Mice were kept in individually ventriculated Typ II cages (IVC, Tecniplast GmbH, Hohenpeißenberg, Germany) in groups of 5 mice, in a 12-h light/dark cycle, with RT at 22 ± 2 °C and a relative air humidity of 45–65%. All mice were allowed free access to water and a maintenance sterile diet. All reasonable efforts were made to ameliorate suffering, including isolation of affected mice. Mice were monitored daily for signs of pain or distress, and repeatedly for body weight. Moribund mice were humanely sacrificed as described below. Study design and biometric planning of each experiment was performed in accordance with a biostatistician. Mice were sacrificed for sample preparation by deep anaesthesia via CO_2_ inhalation followed by cervical dislocation. For each experiment, the single animal was an experimental unit.

For xenograft transplantations, 1 × 10^7^ A549 cells or SK-LU1 cells mixed in matrigel or SK-N-MC cell without matrigel were injected subcutaneously in the flanks of 8–12 weeks old female CD1-nude mice. When the tumours reached a size of 50–150 mm³, systemic treatment with mAB-siRNA-complexes at 4 mg/kg were applied intraperitoneally (i.p.) three times per week in NSCLC and two times per week for Ewing sarcoma. Xenograft tumour size determined the antitumour effectivity. Tumour growth was followed with caliper measurements and tolerance for the animal was assessed by measuring body weight and scoring overall appearance. Tumour volumes were calculated by the formula length × width^2^ × 0.52. At the end of the experiment, animals were euthanized, tumours were isolated, and tumour weight was determined.

### Immunostainings of xenograft tumours

For immunostaining, the A549 tumours were embedded in cryomatrix and SK-LU1 tumours in paraffin using standard methods. Apoptotic cells were detected using TUNEL Assay Kit—HRP-DAB (Abcam cat. no. ab206386, Cambridge, UK) according to the manufacturer’s recommendations.

For Ki67 immunofluorescence, dehydrated paraffin sections were boiled for 60 min in 10 mM citric acid, 0.01% Tween 20, pH 6.0, washed twice in TBS and blocked in blocking solution (2% normal horse serum, 0.1% Tween 20 in TBS). Cryosections were washed three times in TBS/0.1% Tween 20. All sections were incubated with Ki67 antibody (rabbit mAb clone D2H10, cat. no. 9027, Cell Signaling Technology, Frankfurt/Main, Germany) diluted 1:300 in blocking solution overnight at 4 °C, washed three times in TBS/0.1% Tween 20 for 5 min, incubated with secondary antibodies (goat anti-rabbit-Alexa594 diluted in blocking solution for 1–2 h at RT, counterstained with Hoechst 33342 for 5 min in TBS, washed three times with TBS/0.1% Tween 20 and mounted using Dako mounting medium.

### Statistical analysis

All data are presented as means ± standard deviation or standard error, if not indicated otherwise. The mean values of two groups were compared by Student’s *t* test. All *p* values are representing two-tailed analysis.

## Supplementary information

Baeumer Supplementary material
