# Supplementary material for: Targeted siRNA nanocarrier: a platform technology for cancer treatment
Source: Oncogene. 2022 Feb 26;41(15):2210–24. doi: 10.1038/s41388-022-02241-w (PMC8993695; doi:10.1038/s41388-022-02241-w)
Supplement: Supplementary file 1 — Baeumer Supplementary material [file 41388_2022_2241_MOESM1_ESM.docx]

**Supplementary Figures:**

**Targeted siRNA nanocarrier: a platform technology for cancer treatment**

Nicole Bäumer^1^, Jessica Tiemann^1^, Annika Scheller^1^, Theresa Meyer^2^, Lisa Wittmann^1^, Matias Ezequiel Gutierrez Suburu^3^, Lilo Greune^2^, Matthias Peipp^4^, Neele Kellmann^1^, Annika Gumnior^1^, Caroline Brand^1^, Wolfgang Hartmann^5^, Claudia Rossig^6^, Carsten Müller-Tidow^7^, Dario Neri^8^, Cristian A. Strassert^3^, Christian Rüter^2^, Petra Dersch^2^, Georg Lenz^1^, H. Phillip Koeffler^9^, Wolfgang E. Berdel^1^, Sebastian Bäumer^1,^ *


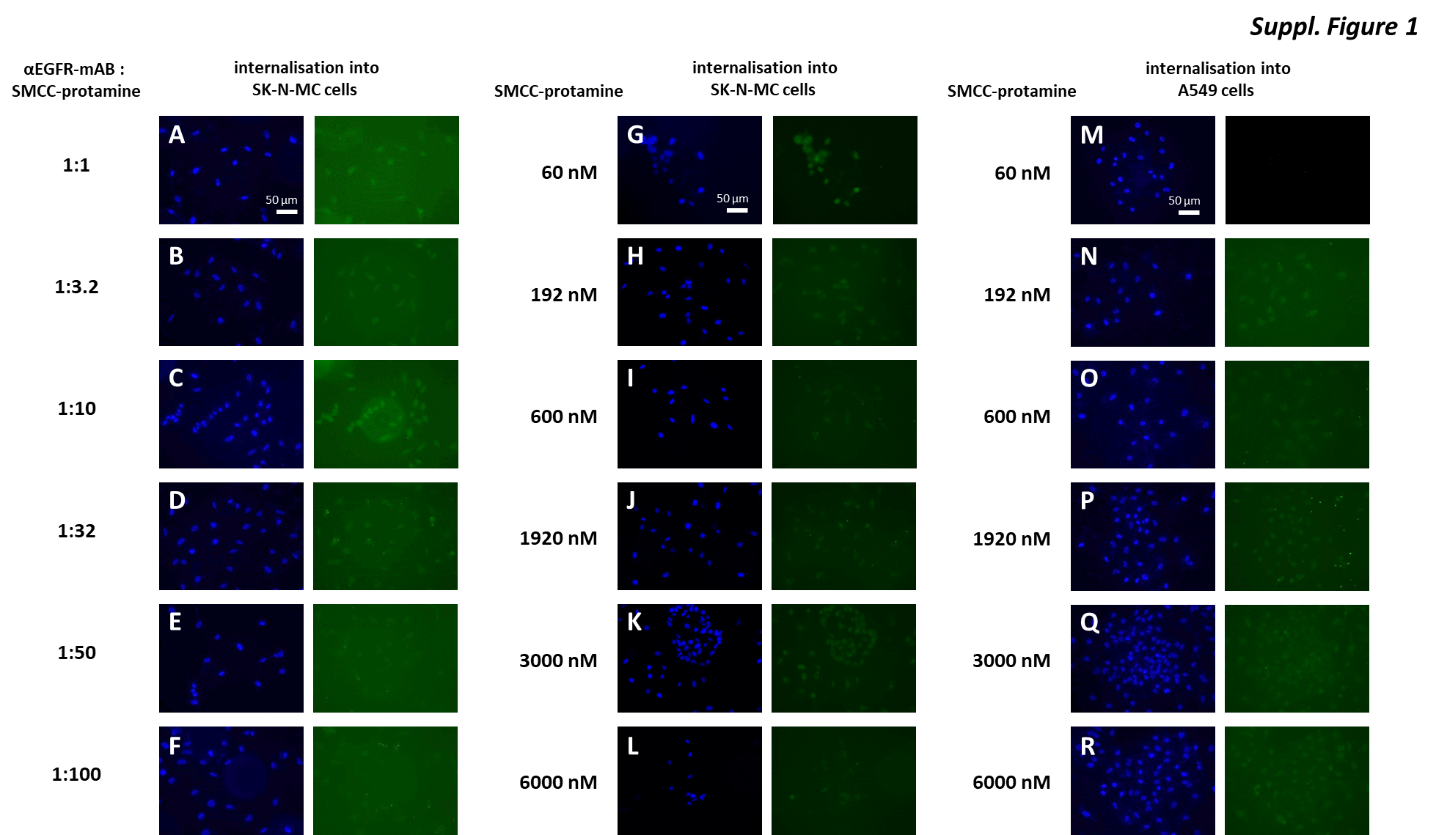


**Supplementary Figure 1: Significance of the targeting antibody within the αEGFR-mAB-protamine/free protamine-siRNA nanoparticle.** **A-F**: The antibody was conjugated to SMCC-protamine as indicated and then coupled to 10x excess Alexa488-siRNA per Mol of antibody and free SMCC-protamine and applied to EGFR-negative SK-N-MC cells. Although the contrast ratio has been exaggerated to the maximum, no internalized Alexa488-siRNA vesicles were seen. The structures in green depict nucleic background staining originating from Hoechst stain. **G-L** and **M-R**: For testing the significance and importance of the targeting antibody both in EGFR-negative SK-N-MC cells and EGFR-positive A549 cells, the targeting antibody has been omitted and the concentrations of protamine-SMCC has adjusted according the mixing ratios in A-F. Also here, there is no internalization of Alexa488 siRNA, which is comparable to results from the complete, targeted αEGFR-mAB-P/P-siRNA nanoparticle in A549 cells in Figure 1, only in the concentration of 1.92 µM SMCC-protamine (P), very few internalized vesicles were detectable. Scale bars: 50 µm.


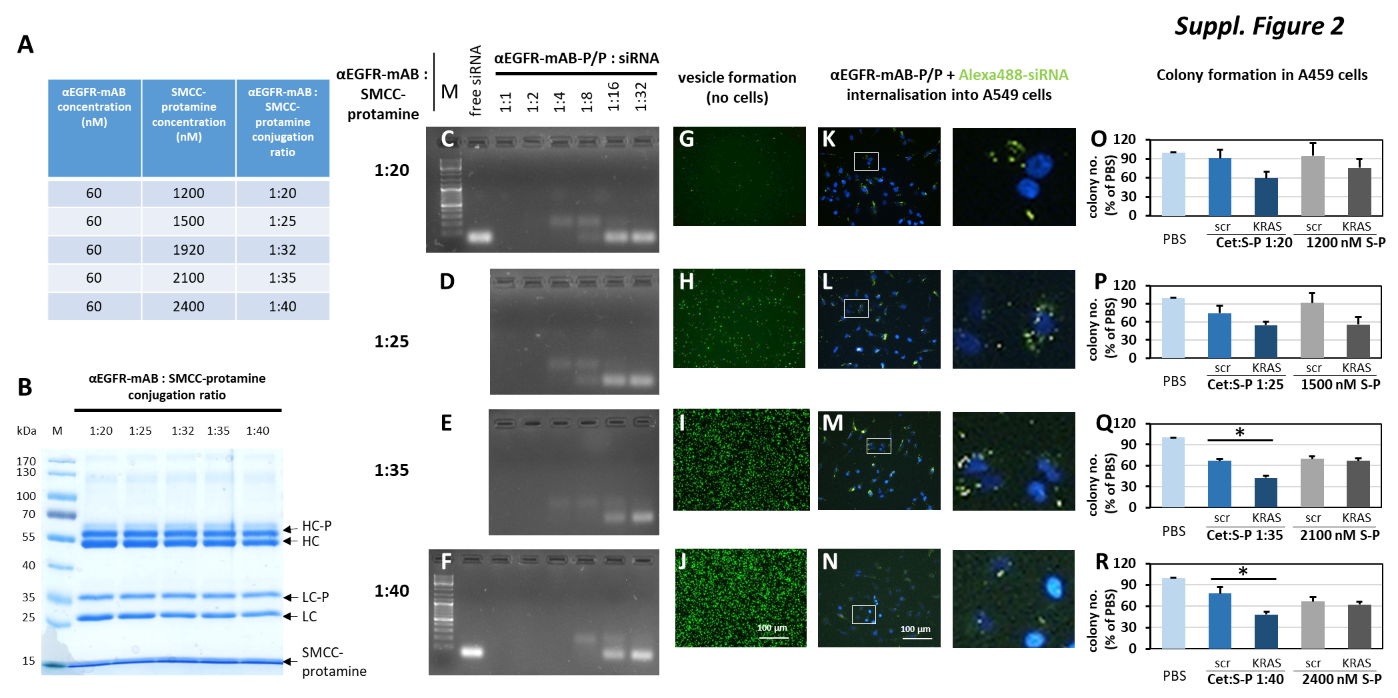


**Supplementary Figure 2: Additional attributes of effective anti-EGFR-mAB-protamine conjugation ratios.** **A.** Concentrations tested in complement to Figure 1 and resulting molar ratios of anti-(α)EGFR antibody (αEGFR-mAB) cetuximab to SMCC-protamine for the effective conjugation of both components. **B.** Coomassie-stained SDS-PAGE showing αEGFR-mAB cetuximab conjugation products that were coupled as depicted in A. The formation of a protamine-conjugated heavy chain (HC-P) and light chain (LC-P) is comparable at all ratios depicted here. **C-F.** Band-shift assays exhibiting siRNA binding capacity. **G-J.** αEGFR-mAB conjugated with rising excess of free SMCC-protamine ranging from 1:20 molar ratio to 1:40 excess of SMCC-protamine in chamber slides (see Fig. 1 A for reaction details). Resulting conjugates were used to bind siRNA in a cell-free standardised assay. The 1:35 (I) and 1:40 (J) ratio mAB to SMCC-protamine formed a homogeneous population of stable particles, whereas the 1:20 (G) conjugates were incompetent and 1:25 (H) less efficient to form stable particles. **K-N.** Internalization of Alexa488-control-siRNA complexed αEGFR-protamine with free SMCC-protamine (αEGFR-mAB-P/P) in A549 cells. Complexes of αEGFR-mAB-P/P transport Alexa488-siRNA into cells (left panel rectangles), with detailed magnifications (right panels). **O-R.** Colony formation assays using the complexes analysed in C-F, G-J, and K-N in EGFR-positive A549 cells. Significant (*) effects of αEGFR-mAB-P/P transported KRAS siRNA effect in contrast to control scrambled (scr) siRNA are only seen in conjugate preparations with 1:35 (Q) and 1:40 (R) molar ratio mAB to protamine. Conversely, lower ratios show no sufficient functional effect (**O-P**), while preparations with higher molar excess of protamine-SMCC show toxicity independent of KRAS knockdown (**Q-R**). Cet:S-P, αEGFR-antibody cetuximab conjugated to SMCC-protamine at the indicated ratios; S-P, SMCC-protamine. Mean +/- SD of 3 independent experiments. 2-sided t-test, * p < 0.05. α, anti.


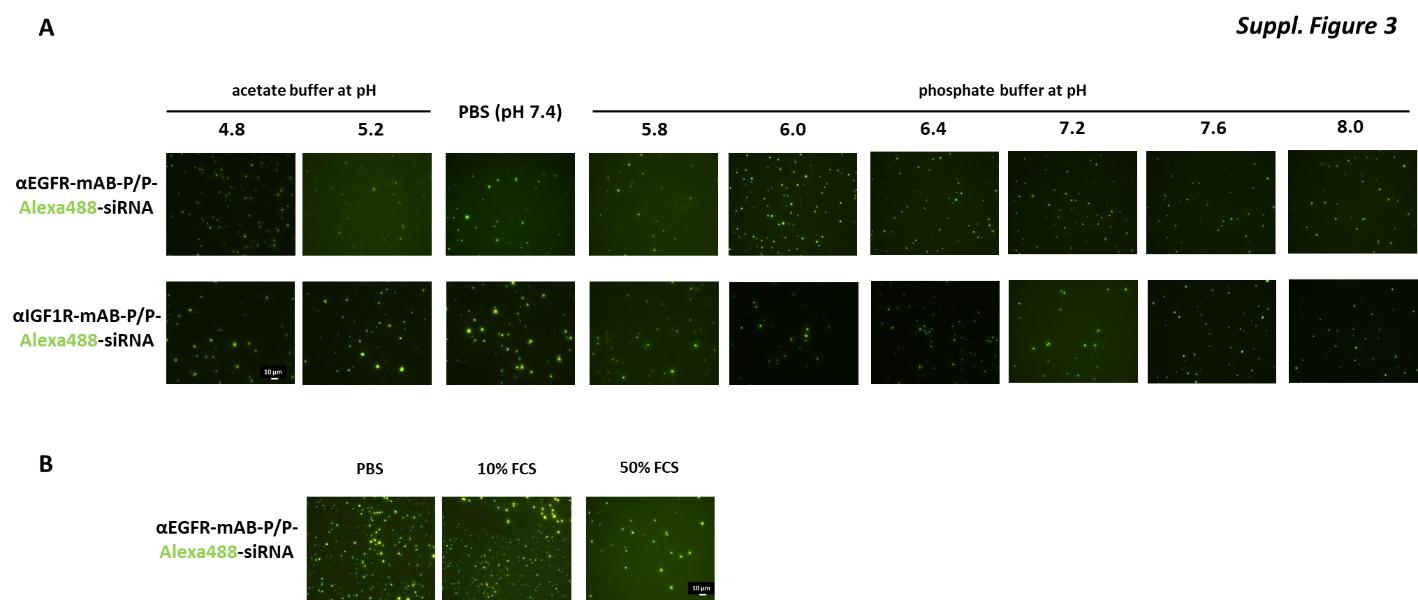


**Supplementary Figure 3: Fluorescence microscopy to determine the stability of nanocarriers in different conditions.** Stability after 2 h-auto-assembly of αEGFR-mAB-P (**A**, upper panels, and **B**) or αIGF1R-mAB-P (**A**, lower panels), in presence of 32x free protamine and Alexa488-siRNA in a 1:10 ratio and subsequent incubation for 24 h in (**A**) buffers of the indicated pH or (**B**) cell culture medium RPMI/10% FCS and PBS/50% FCS compared to PBS. α, anti.


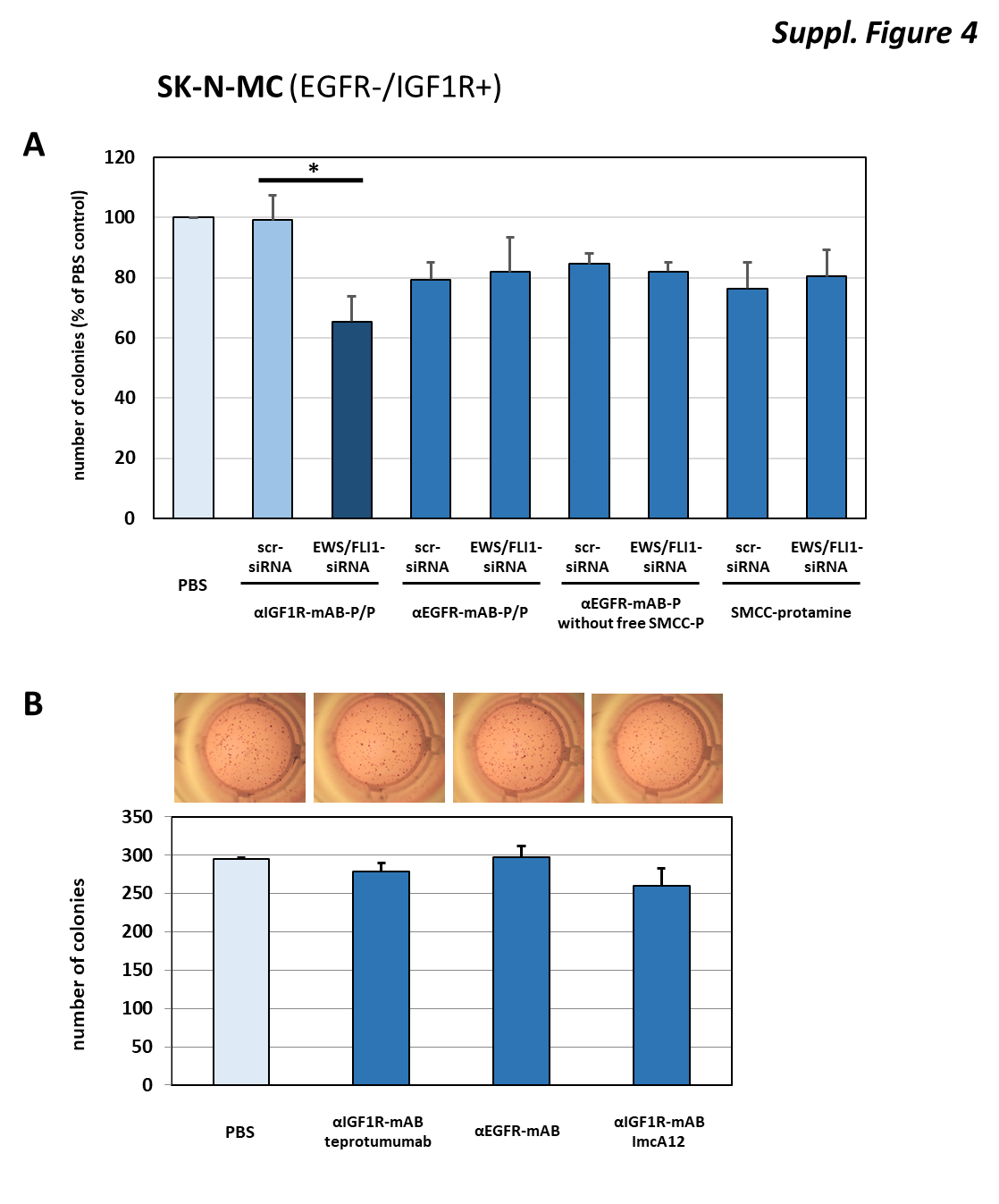


**Supplementary Figure 4: Significance of the targeting antibody of the mAB-protamine-siRNA-protamine nanocarrier tested by functional colony formation assay.** **A.** EGFR-negative and IGF1R-positive Ewing sarcoma cells SK-N-MC were treated with αIGF1R-mAB-P (αIGFR-mAB-P), αEGFR-mAB-P and αEGFR-mAB-P that passed a protamine-depletion step, as well as unconjugated SMCC-protamine, all at 60 nM end concentration. Only the relevant αIGF1R-mAB-P plus free SMCC-protamine in conjunction with the relevant EWS/FLI1 siRNA reached a detectable and significant reduction of colony formation to 63% of the control. All other controls showed no major reduction of the colony formation. **B.** Colony formation in SK-N-MC cells treated with 60 nM unconjugated αIGF1R-mAB teprotumumab, unconjugated αEGFR-mAB, or αIGF1R-mAB ImcA12, respectively, is unchanged compared to PBS treated cells. Significance: * p < 0.05 (t-test, two-tailed). Please refer to Figure 7 for further results. α, anti.


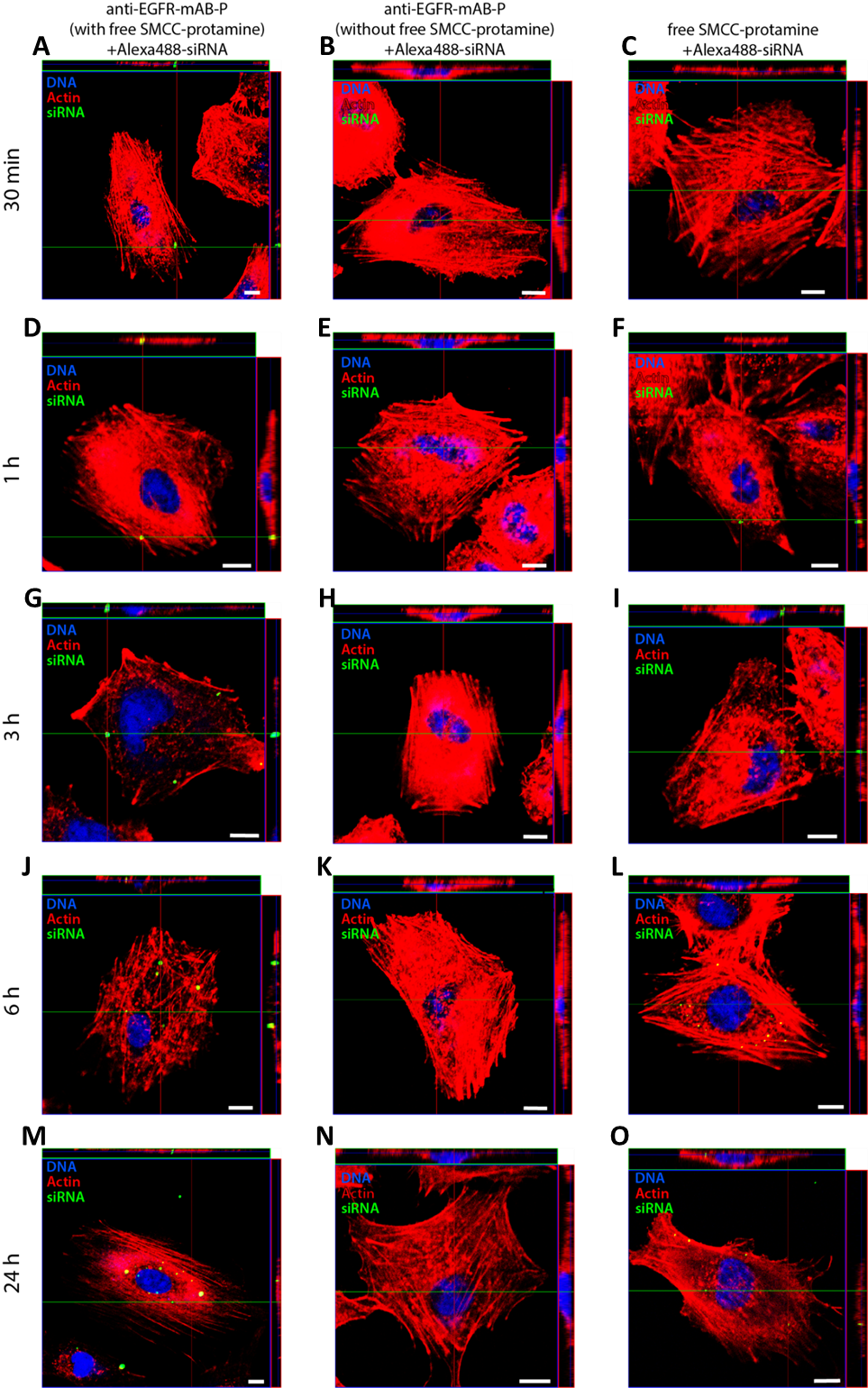


**Supplementary Figure 5: Enrichment of internalized Alexa488-siRNA vesicles by αEGFR-mAB-nanoparticles in EGFR-positive A549 cells.** Over a time course of 30 minutes to 24 hrs, a clear accumulation of Alexa488-siRNA containing intracellular vesicles were detected (**A, D, G, J, M**). An αEGFR-mAB-P that passed a depletion step of free SMCC-protamine was not able to show similar internalization activities (**B, E, H, K, N**). Un-targeted SMCC-protamine in order to force an un-targeted transfection of siRNA (**C, F, I, L, O**) revealed a number of very small green fluorescent intracellular vesicles, which were non-functional as seen by Figure 1 R. Scale bars 10 µm.


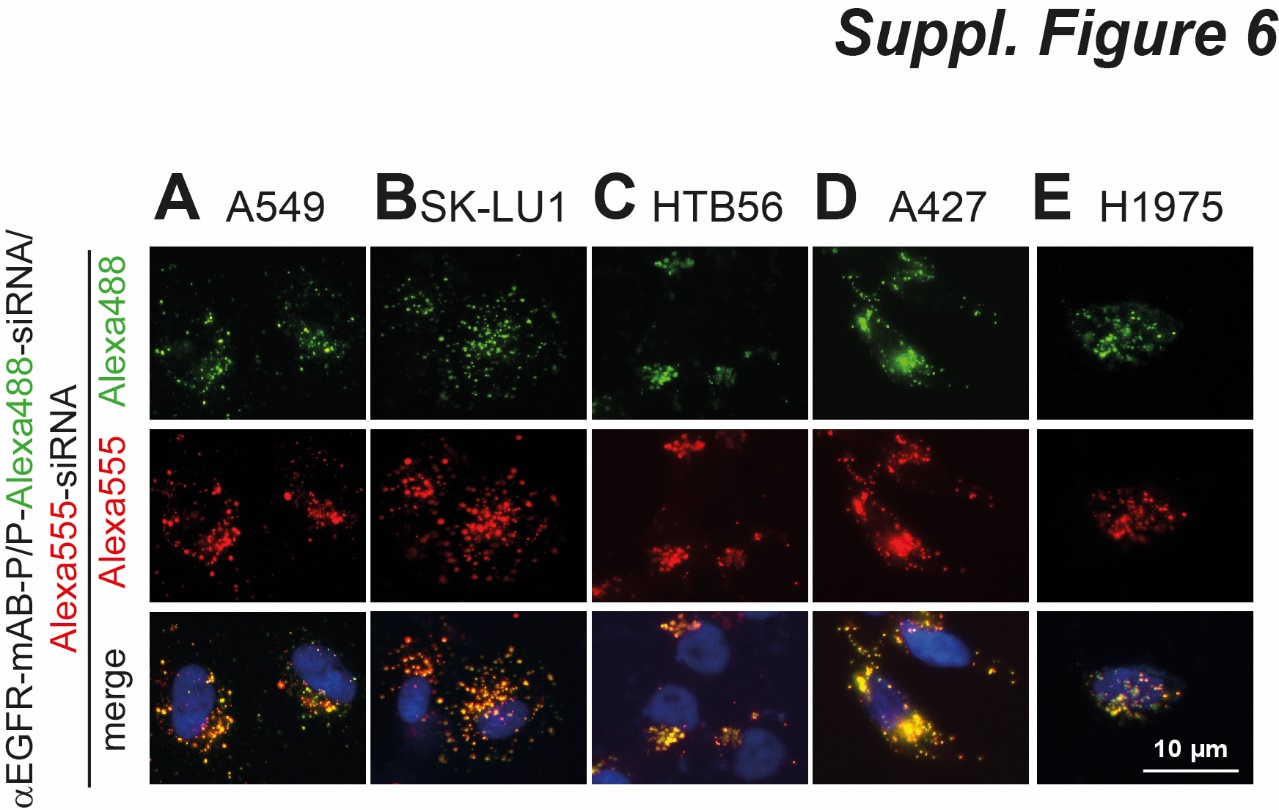


**Supplementary Figure 6: Impact of a combined use of αEGFR-mAB nanostructures carrying two different siRNA in NSCLC models.** **A-E.** Five further different NSCLC cell lines were treated with the nanostructures described carrying a combination of two siRNAs tagged with green Alexa488 and red Alexa555. All EGFR-expressing NSCLC cell lines were hit by both siRNAs resembling convergent intracellular vesicular structures, indicating that a combined therapy targeting two oncogenes is possible. α, anti.

**
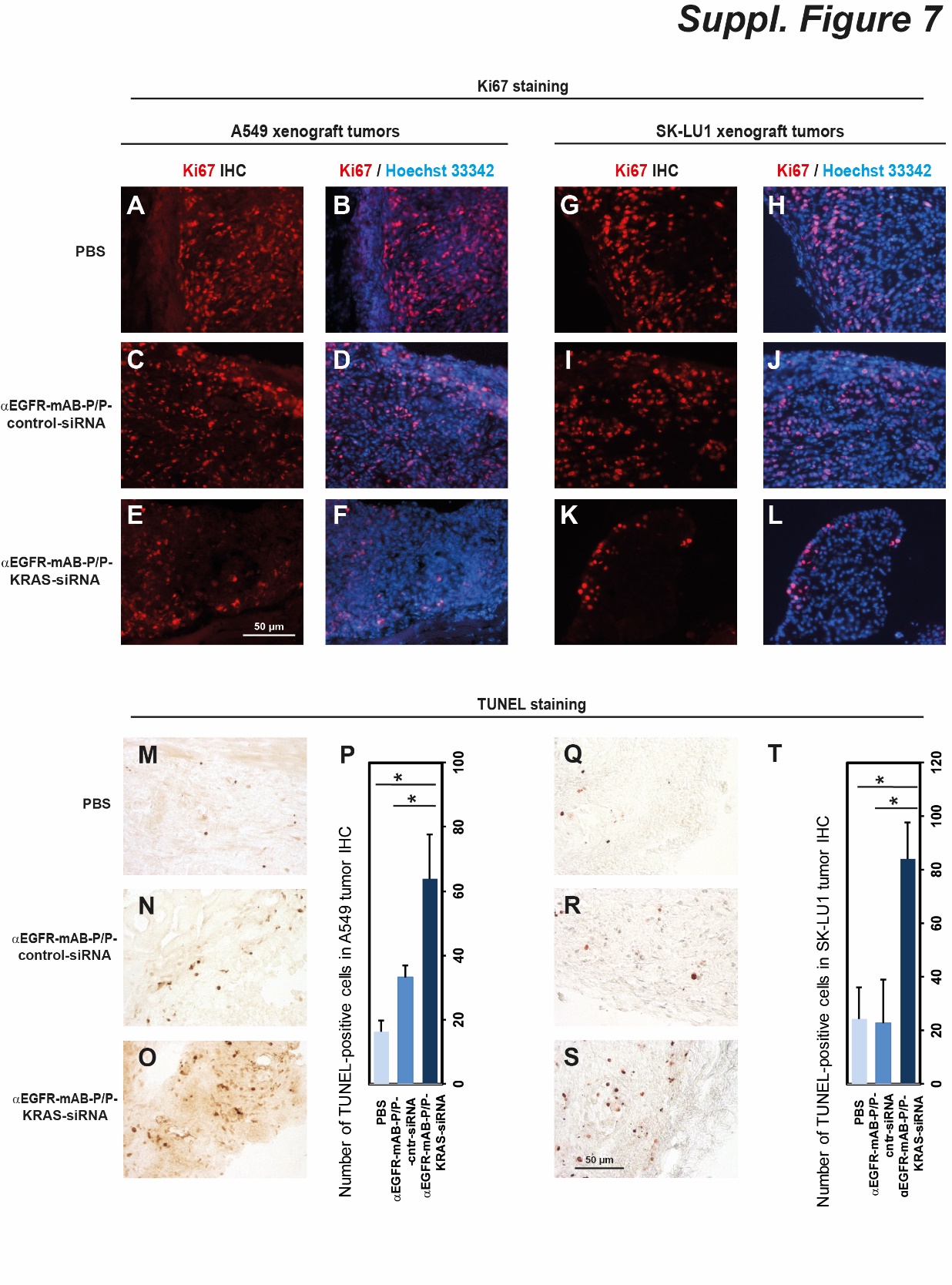
Supplementary Figure 7: Proliferation marker Ki67 and apoptosis determined *ex vivo* in NSCLC xenografts upon systemic treatment with nano-carriers.** Immune histology fluorescence (IHC) staining of proliferation marker Ki67 on histological xenograft sections. Compared to the PBS and control siRNA nano-carrier treated groups, the number of Ki67 positive nuclei were massively reduced in KRAS-siRNA nanocarrier treated tumor histological sections in A549 **(A – F)** as well as in SK-LU1 **(G – L).** Immunohistological determination of apoptosis in xenograft tumor sections by TUNEL assay. **M-T.** A significantly increased rate of apoptosis was seen in KRAS-siRNA nano-carrier treated tumors as compared to control groups in both xenografted cell lines. **P, T.** Statistics of TUNEL-positive nuclei in sections: The number of TUNEL-positive nuclei was two-fold increased in A549 tumor treated with αEGFR-mAB-P/free protamine (P) nanoparticles when compared to PBS treatment and three-fold increased within tumors treated with KRAS-siRNA nano-carriers. In SK-LU1, only αEGFR-mAB-P/free protamine-KRAS-siRNA nano-carrier treatment led to a four-fold increase of apoptotic cells. cntr, control. Significance: * p < 0.05, 2-sided t-test. α, anti.

**
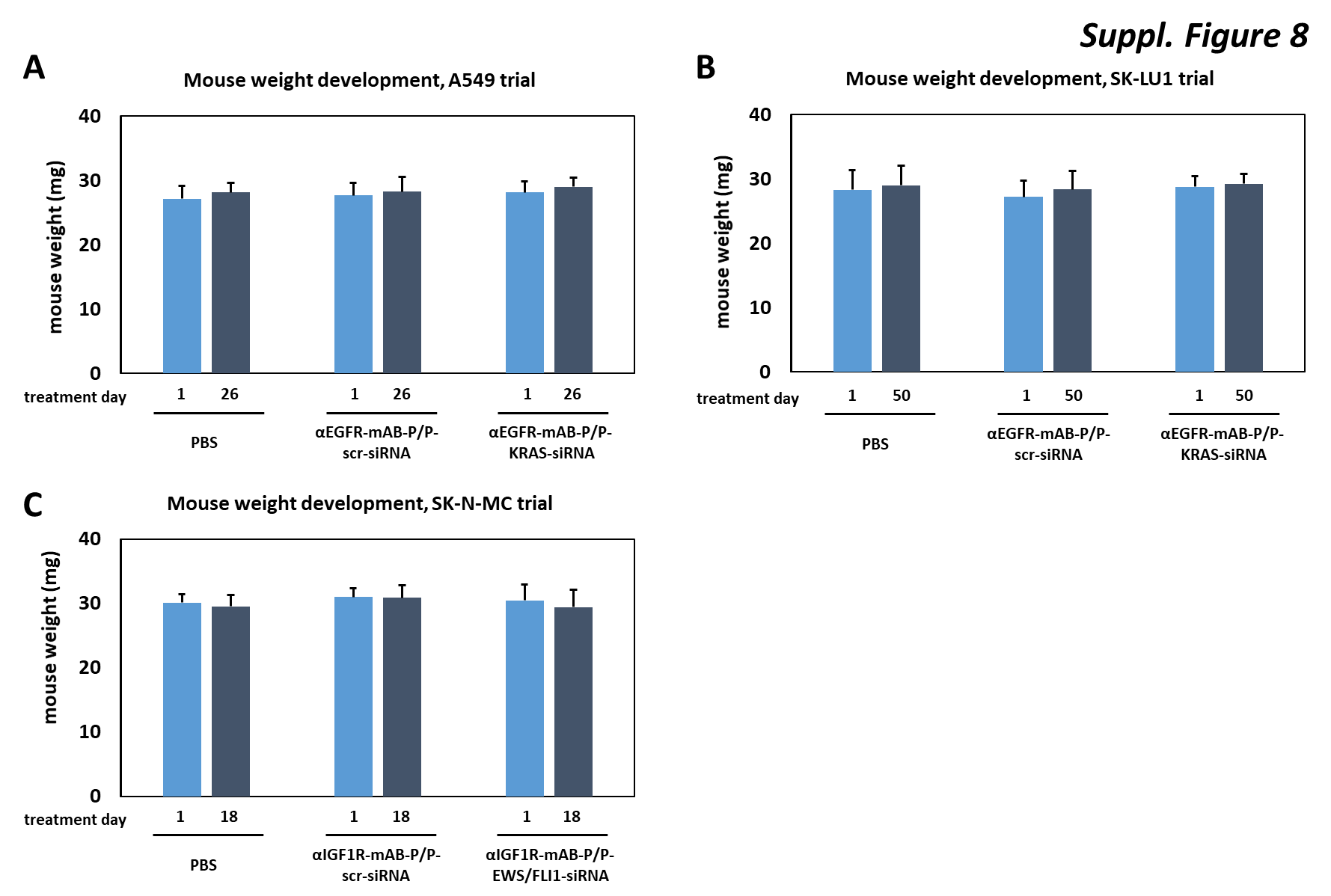
**

**Supporting Figure 8: Determination of toxicity parameters in transplanted and treated mice.** In the *in vivo* experimental trial presented in Figure 6 B (A549 cells), Figure 6 C (SK-LU1 cells) and Figure 8 D (SK-N-MC cells), respectively, mouse weight values were determined on each treatment day. Shown here are the values of day one and after the treatment on day 26 (**A**), day 50 (**B**) and day 18 (**C**). The values varied insignificantly in all groups exposing no obvious signs of toxicity.
